# Supplementary material for: The 20S as a stand-alone proteasome in cells can degrade the ubiquitin tag
Source: Nat Commun. 2021 Oct 26;12:6173. doi: 10.1038/s41467-021-26427-0 (PMC8548400; doi:10.1038/s41467-021-26427-0)
Supplement: Supplementary file 1 — Supplementary Information [file 41467_2021_26427_MOESM1_ESM.pdf]

Supplementary Information for:

The 20S as a stand-alone proteasome in cells can degrade the ubiquitin-tag

Indrajit Sahu[1], Sachitanand M. Mali[2], Prasad Sulkshane[1], Cong Xu[3], Andrey Rozenberg[1], Roni Morag[1], Manisha Priyadarsini Sahoo[1], Sumeet K. Singh[2], Zhanyu Ding[3], Yifan Wang[3], Sharleen Day[4], Yao Cong[3,5], Oded Kleifeld[1,*], Ashraf Brik[2,*], Michael H. Glickman[1,*]

*Correspondence to: glickman@technion.ac.il, okleifeld@technion.ac.il, or abrik@technion.ac.il

This PDF file includes (in the following order):

Supplementary Figs. 1 to 21

Supplementary Tables 1 to 4

Supplementary Methods with Supplementary Figs. 22 to 33

Additional Supplementary information for this manuscript can be found in the following files:

Supplementary Data 1 to 11

Supplementary Data descriptions correspond to Supplementary Data 1 to 11

Supplementary Figures

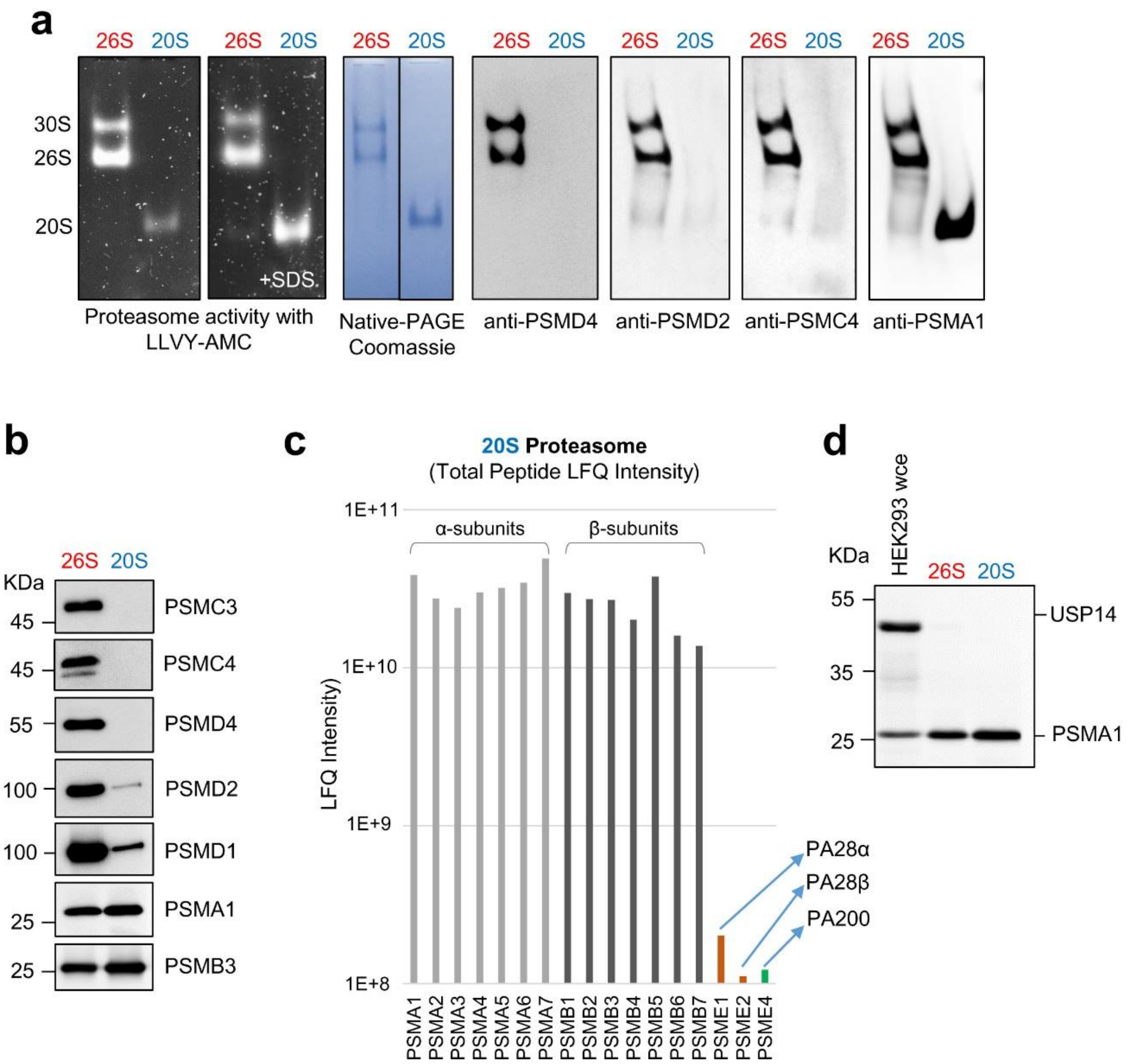

Supplementary Fig 1: Purity and integrity of purified human proteasomes. a, Purified 26S and 20S proteasome samples were resolved by 4% non-denaturing PAGE (native gel), checked for in-gel activity with LLVY-AMC peptides, and immunoblotted with different proteasome subunit antibodies. **b,** Purified 26S and 20S proteasome samples were separated by 12% SDS-PAGE and immunoblotting was performed using different proteasome subunit antibodies as indicated. **c**, A purified 20S proteasome sample was subjected to LC-MS/MS to identify the subunit composition. The bar graph summarizes the total peptide LFQ intensity of each α, β subunit in the sample and the trace amounts of other related subunits identified in the sample.

The total peptide LFQ intensity was normalized to the copy number of each subunit within the 20S complex. **d**, An immunoblot confirming the absence of detectable USP14 in the purified 26S or 20S proteasome samples. We noted that neither USP14 nor UCHL5 were detected in the 20S sample by MS/MS. HEK293T cell lysate was taken as a positive control for USP14. Source data are provided as a Source Data file.

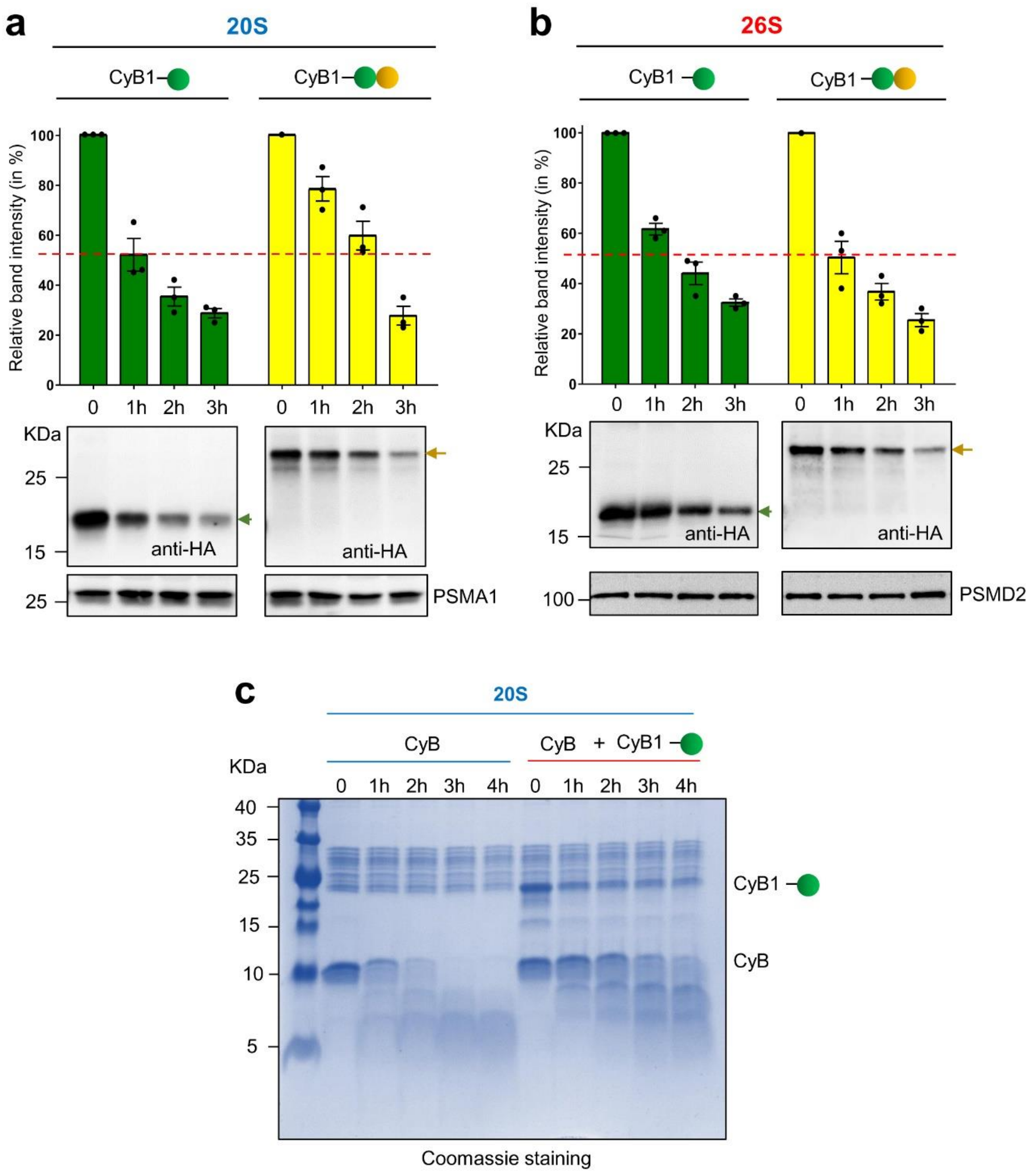

Supplementary Fig 2: Proteolysis of ubiquitinated CyclinB1-NT by 20S or 26S proteasomes. MonoUb-CyclinB1-NT or DiUb-CyclinB1-NT were incubated separately with either **a**, purified 20S, or **b**, 26S proteasome at 1:150 (Proteasome:Substrate) molar ratio for indicated time periods at 37ºC. Each reaction mixture was resolved by SDS PAGE and remaining substrate traced by IB (lower panel) and residual protein quantified by ImageJ. The bar graph summarizes the values (±SEM error bar) quantified by anti-HA IB (normalized to

either anti-PSMA1 or anti-PSMD2 IB) from three independent experiments. **c**, Unmodified CyclinB1-NT was incubated alone (left) or together with equimolar MonoUb-CyclinB1-NT (right) with purified 20S proteasome for indicated time periods at 37ºC. Final ratios were 1:150 and 1:300 (Proteasome:Substrates) molar ratios respectively. Each reaction mixture was resolved by SDS PAGE and stained with Coomassie. Source data are provided as a Source Data file.

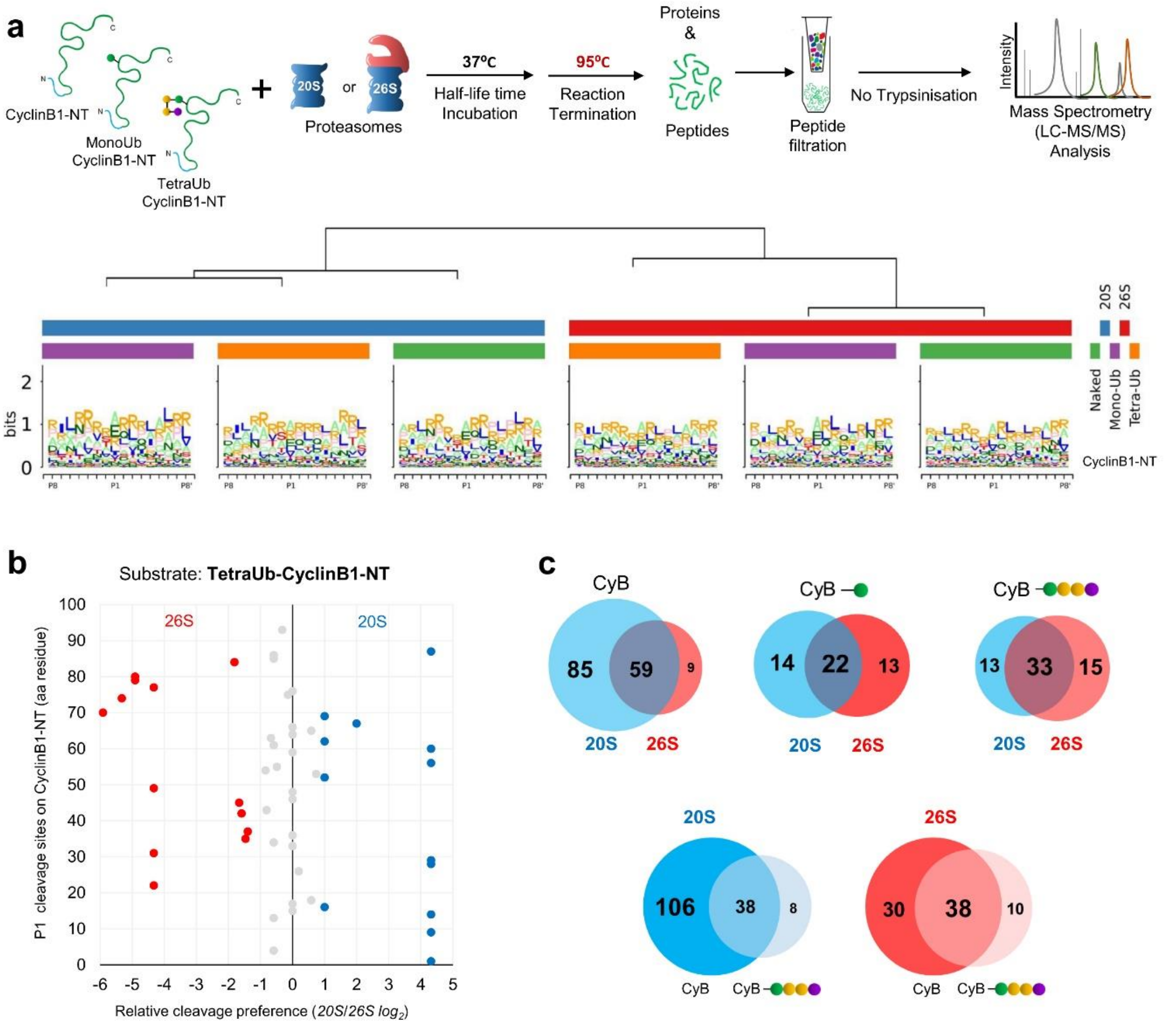

Supplementary Fig 3: LC-MS/MS analysis of peptides generated *in vitro* from synthetic substrates by proteasomes. a, CyclinB1-NT, MonoUb-CyclinB1-NT or TetraUb-CyclinB1-NT were incubated separately at 37ºC with either purified 20S or 26S proteasome at 1:200 (Proteasome:Substrate) molar ratio. Reaction was quenched by snap heating to 95ºC when less than 40% of initial substrate was degraded to minimize potential of reprocessing of peptide products. Peptides were filtered from the reaction mixture, isolated, concentrated, and analyzed by LC-MS/MS. Sequence logo summarizes the amino acid preference for P1 cleavage sites and neighboring sequences up to 8 residues to either side on each substrate for each of the two proteasome species tested. **b**, Scatter plot represents the relative cleavage preference for each proteasome species at P1 positions on TetraUb-CyclinB1-NT. Cleavage preference for each

enzyme was calculated from the MS/MS count of each peptide contributing to a given P1 site and the relative ratio plotted as $\log_2$ (described in Methods). The Y-axis represents the amino acid residue number of the HA-CyclinB1-NT sequence. Red/Blue dots indicate a ≥2 fold preference (Red, 26S; Blue, 20S; grey, no significant preference). **c**, Venn diagrams represent the unique and common peptides generated by each proteasome from the given substrates.

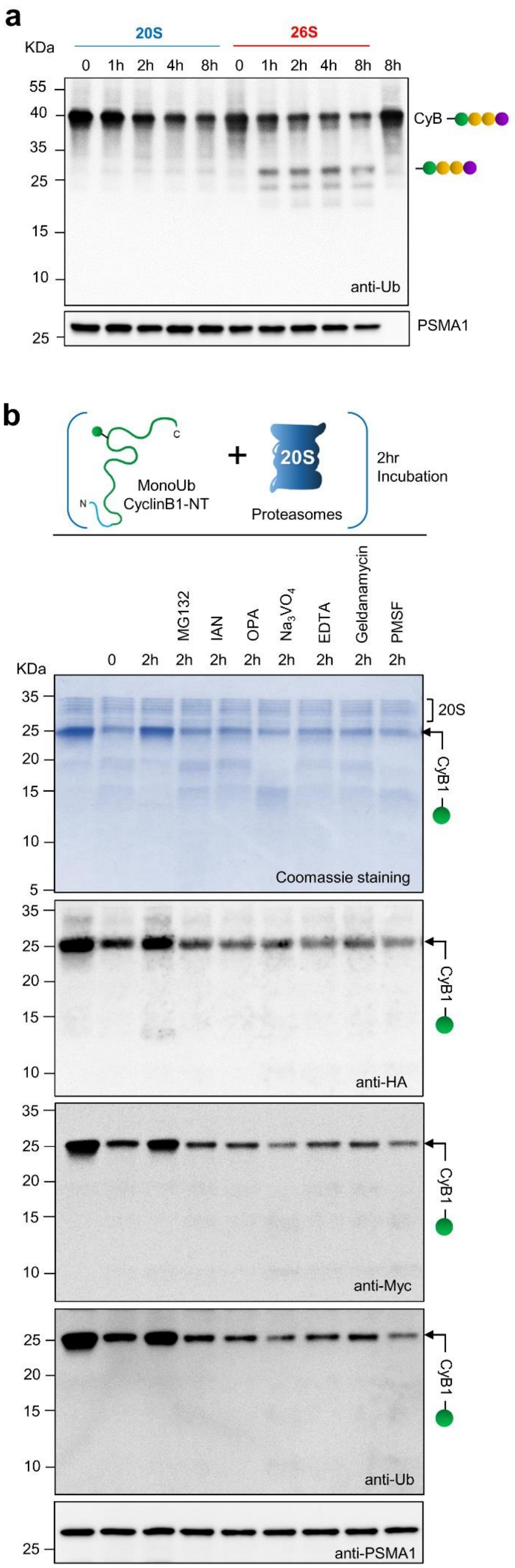
a
20S
26S
KDa
0 1h 2h 4h 8h 0 1h 2h 4h 8h 8h
55
40
35
25
15
10
CyB
anti-Ub
25
PSMA1
b
MonoUb
CyclinB1-NT
N
C
20S
Proteasomes
2hr
Incubation
MG132
IAN
OPA
Na_3VO_4
EDTA
Geldanamycin
PMSF
0 2h 2h 2h 2h 2h 2h 2h 2h
KDa
35
25
20
15
10
5
20S
CyB1
Coomassie staining
anti-HA
anti-Myc
anti-Ub
anti-PSMA1

Supplementary Fig. 4: Inhibitors against possible contaminating proteases do not affect proteolysis by purified 20S proteasome sample. a, TetraUb-CyclinB1-NT was incubated with purified 20S or 26S proteasome at a 1:150 (Proteasome:Substrate) molar ratio for indicated time periods at 37ºC. The reaction mixture was resolved by 14% Tris-Tricine PAGE and probed with anti-ubiquitin antibody. **b**, The Mono(Myc)Ub-(HA)CyB-NT substrate was incubated with 20S proteasome at a molar of ratio 1:150 (Proteasome:Substrate) in 20S-buffer [25 mM Tris (Ph7.4), 150 mM NaCl, 10% Glycerol, 1mM DTT] for the indicated time period at 37°C with or without different inhibitors: MG132 – a 20S proteasome inhibitor; IAN, iodoacetamide – an inhibitor for cysteine-based DUBa; OPA, 1,10(O)-phenanthroline – an inhibitor for Rpn11; Na_3VO_4, sodium ortho-vanadate – an ATPase inhibitor; EDTA – a metal ion chelator (for trace metallo-proteases or as an ATPase inhibitor by neutralizing residual $MgCl_2$); geldanamycin – an HSP90 inhibitor; PMSF – a broad specificity protease inhibitor. The reaction mixture was separated by 14% Tris-Tricine SDS-PAGE followed by either Coomassie staining or immunoblotting with anti-Ub, anti-Myc and anti-HA antibodies. Source data are provided as a Source Data file.

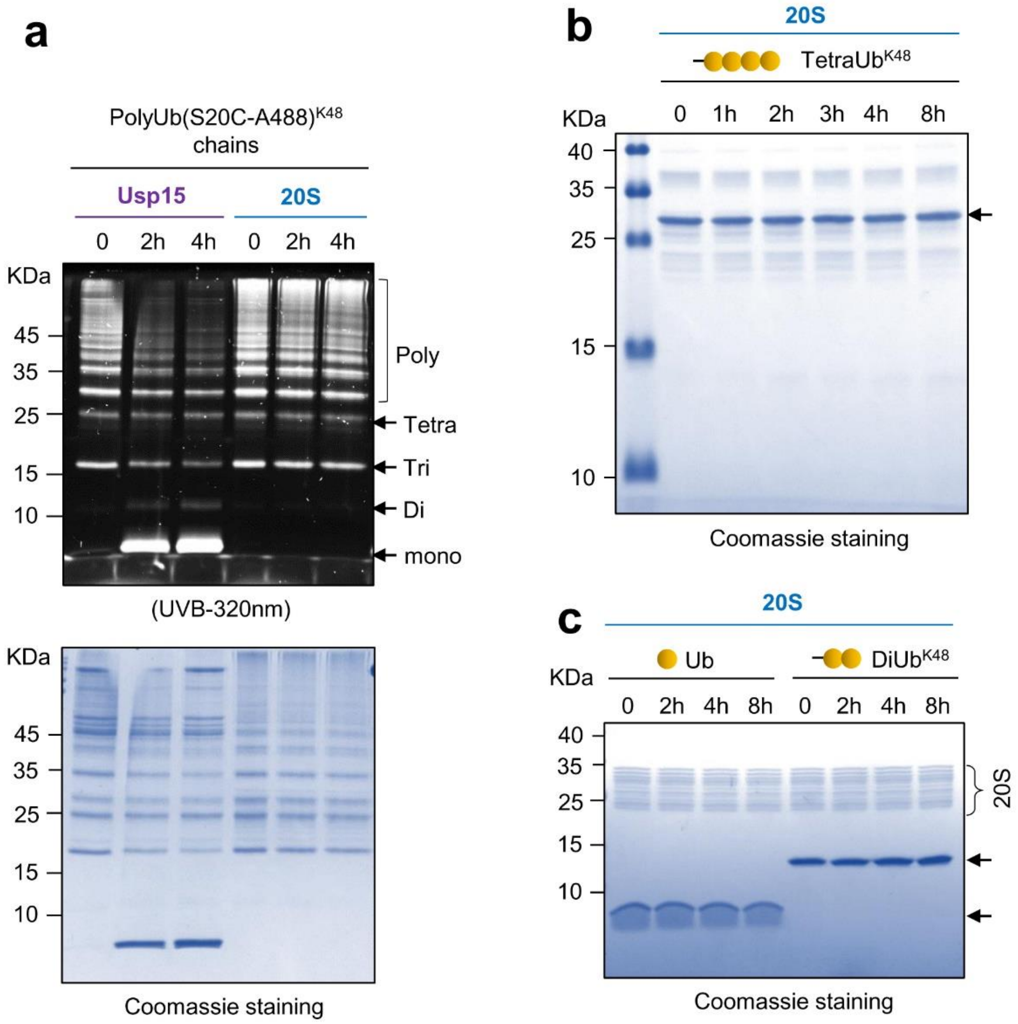

Supplementary Fig. 5: The 20S proteasome neither process unanchored ubiquitin-chains nor degrades them. a, Fluorescently labeled (S20C-FITC) K48-linked polyUb chains were incubated with either 100nM purified 20S proteasomes or 100nM recombinant USP15 (as a control) at 37°C in the DUB buffer [50 mM TRIS (pH 7.4), 50 mM NaCl, 100 µM EDTA, 5 mM DTT] for the indicated time period. The reaction mixture was separated by 15% SDS-PAGE followed by fluorescent imaging and then Coomassie staining. **b**, Free TetraUb^{K48} chains, or **c**, free ubiquitin or DiUb^{K48} chains were incubated at 37ºC with purified 20S or 26S proteasome at 1:150 (Proteasome:Substrate) molar ratio for the indicated time period. The reaction mixture was resolved by Tris-Tricine PAGE and stained with Coomassie. Source data are provided as a Source Data file.

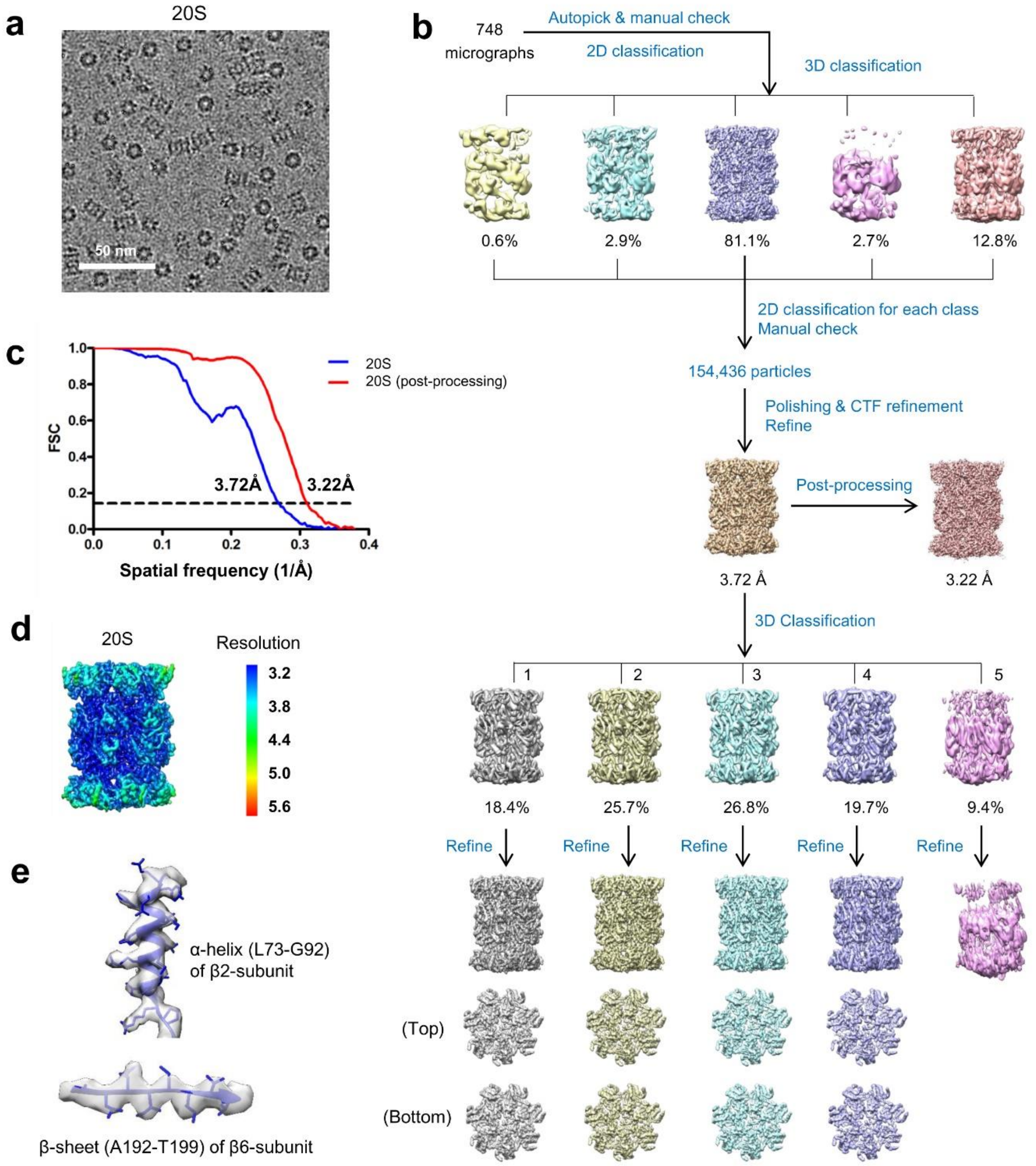

Supplementary Fig. 6: Workflow and validation for the cryo-EM data processing of 20S proteasome complexes. a, Representative cryo-EM micrograph of 20S proteasome. **b**, Micro 3D classification and refinement procedures. After 2D and 3D classifications to eliminate bad particles, there are 154,436 remaining cleaned-up particles, based on which we obtained an overall map for 20S alone sample. Further 3D classification did not yield significant differences between classes for the four classes with reasonably good structural features (1 to 4), and their

two gates all appear symmetrically closed. **c**, Resolution estimation of these obtained maps according to the gold standard FSC criterion of 0.143. **d**, Local resolution estimation for the 20S alone map. The resolution color bar (in Å) is also shown. **e**, Density details for 20S only map before post-processing (3.72Å) at α-helix (L73-G92) of β2-subunit and β-sheet (A192-T199) of β6-subunit.

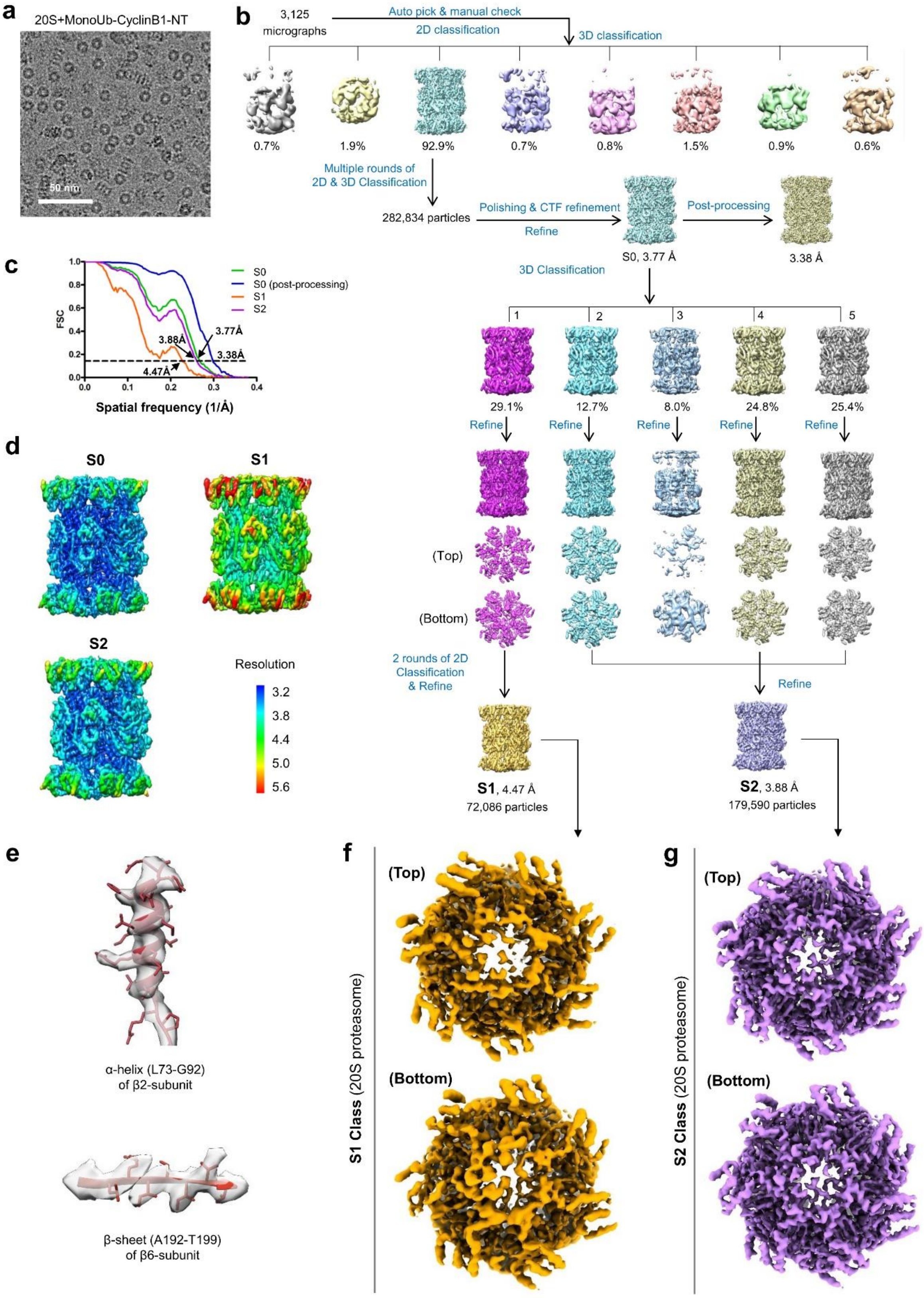

Supplementary Fig. 7: Workflow and validation for the cryo-EM data processing of 20S proteasome incubated with MonoUb-CyclinB1-NT. a, Representative cryo-EM micrograph

of 20S proteasome incubated with MonoUb-CyclinB1-NT. **b**, 3D classification and refinement procedures. After 2D and 3D classifications to eliminate bad particles, there are 282,834 remaining cleaned-up particles, based on which we obtained an overall map for 20S+MonoUb-CyclinB1-NT (named as S0). A further 3D classification into 5 classes generated a class (class 1) with an obviously asymmetric gate configuration (in magenta, 29.1% of the population), which is distinct from the other classes. This class was further processed to generate a map denoted as S1. For the other three classes with reasonably good structural features (2, 4, and 5), since they all appear symmetric between the two rings and show highly similar features, their particles were combined and refined into a map denoted as S2. **c**, Resolution estimation of these obtained maps according to the gold standard FSC criterion of 0.143. **d**, Local resolution estimations for the S0, S1, and S2 maps. Their resolution color bars (in Å) are also shown. **e**, Density details for S1 map (4.47Å) at α-helix (L73-G92) of β2-subunit and β-sheet (A192-T199) of β6-subunit. **f**, Top and bottom view of S1 20S proteasome shows asymmetric gate opening. **g**, Top and bottom views of the map of S2 20S proteasome showing symmetric gate configuration.

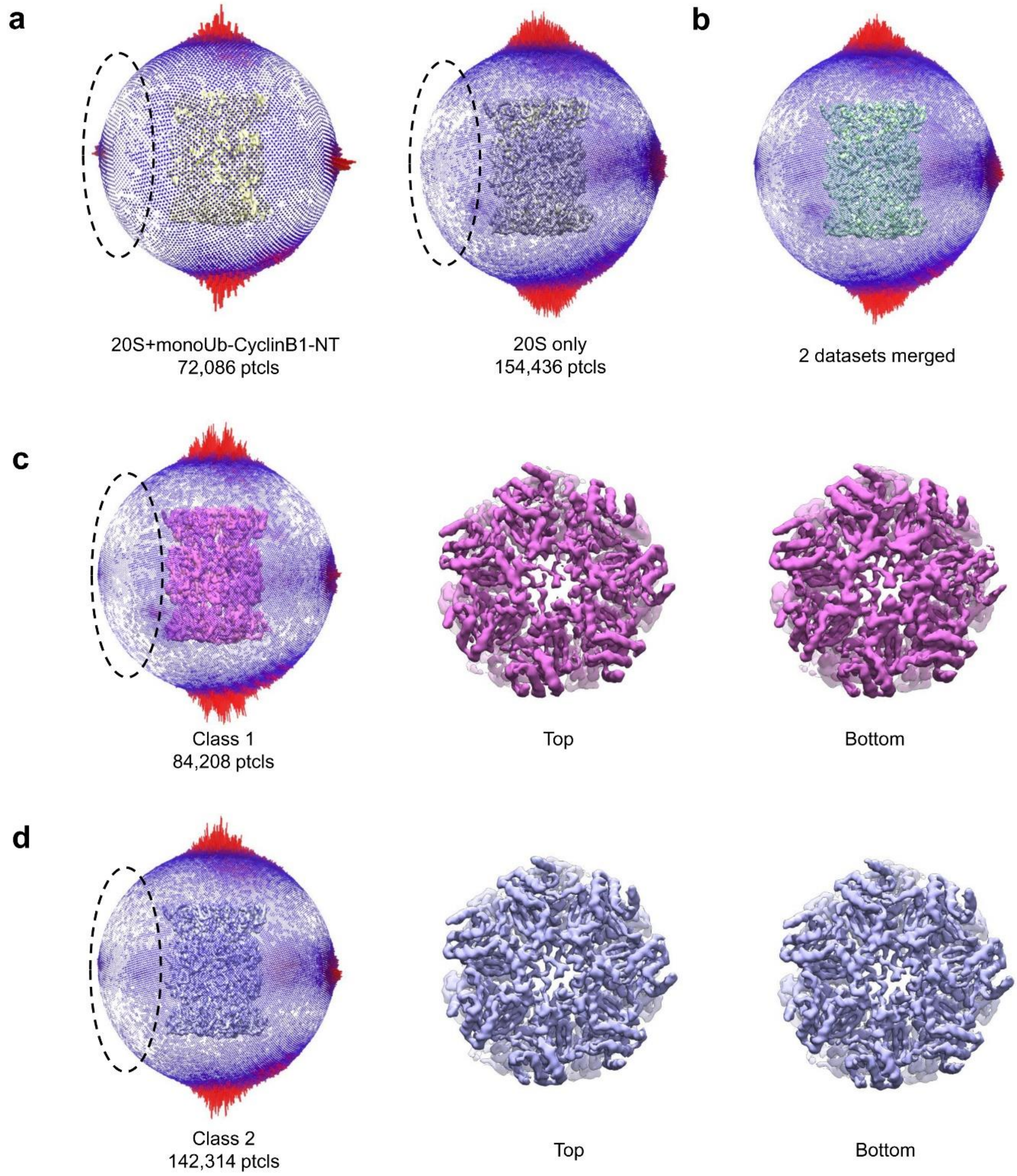

Supplementary Fig. 8: Angular distribution and gate status of the cryo-EM 3D reconstructions. a, Angular distribution plot of the 20S+monoUb-CyclinB1-NT and the 20S only cryo-EM maps. **b**, Angular distribution plot of the map reconstructed from the combined dataset. **c-d**, Angular distribution plot and close up views of the two α-rings for the Class1 c, and Class 2 d, maps, which are 3D classified from the combined dataset and further auto-refined.

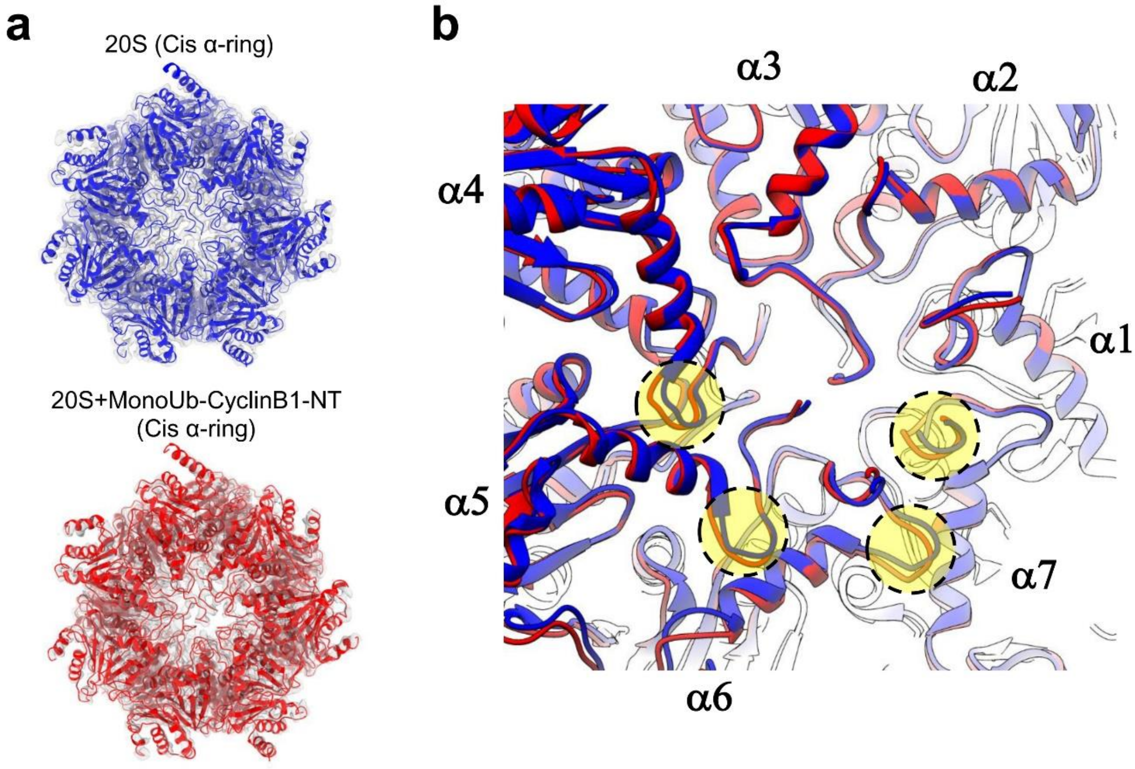

Supplementary Fig. 9: Comparison of 20S models. (**a**) Model-map fitting of 20S only (top) and 20S+monoUb-CyclinB1-NT (bottom) structures. The maps have been processed by DeepEMhancer. (**b**) Overlapping of the two models (20S+monoUb-CyclinB1-NT in red; 20S only in blue) highlighting changes to the reverse turn regions (shaded yellow).

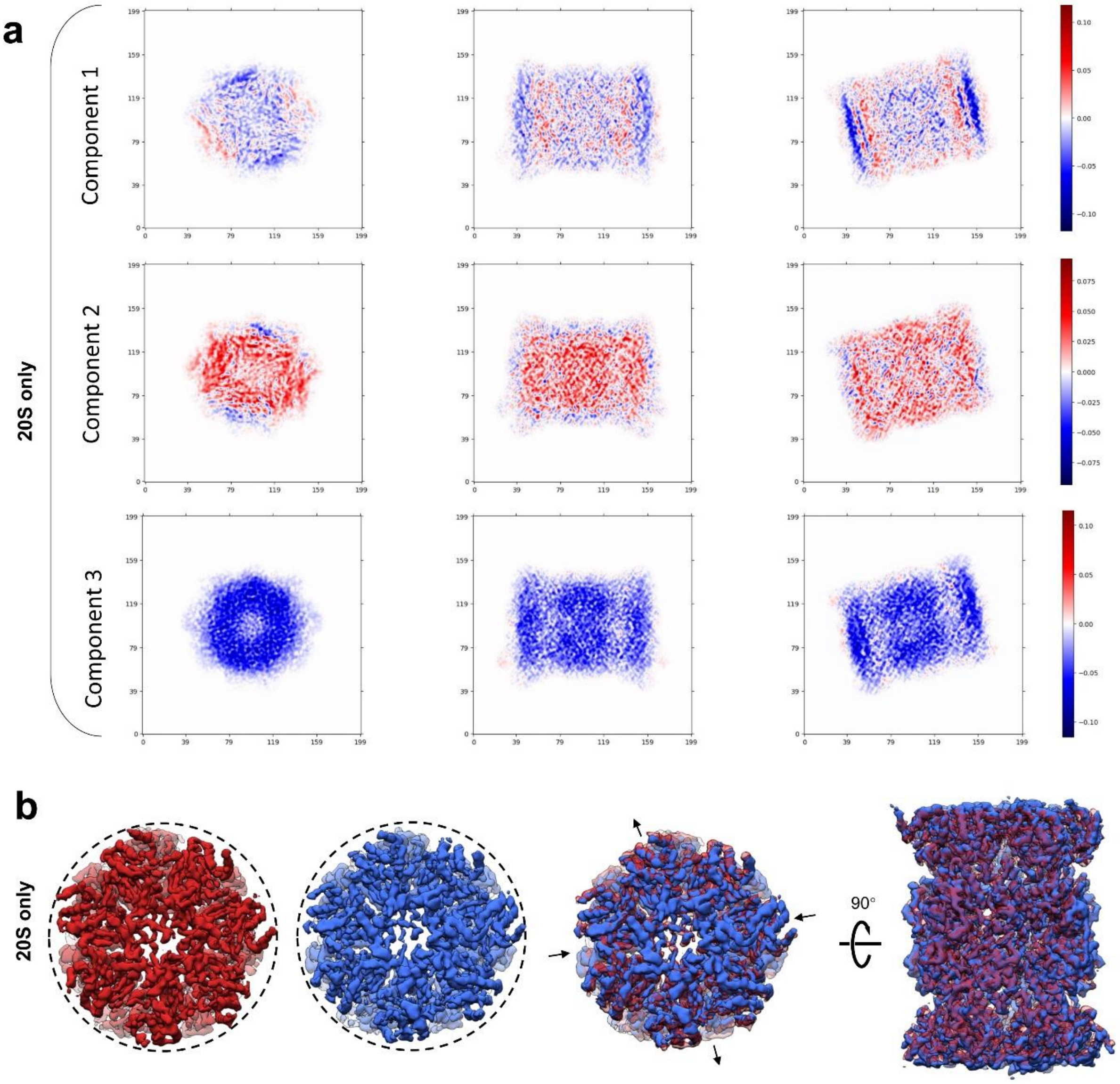
a
20S only
Component 1
Component 2
Component 3
b
20S only
90°

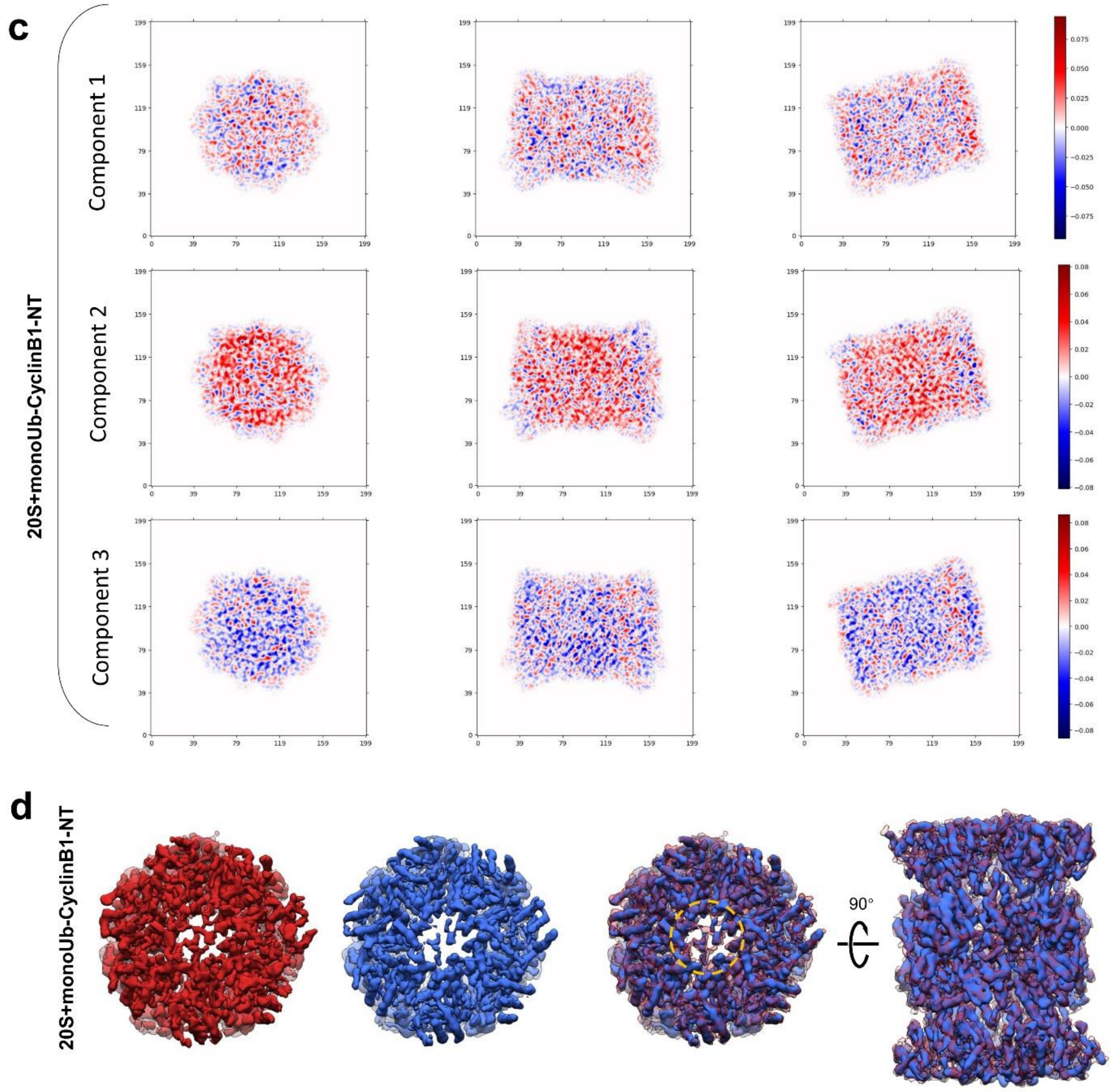

Supplementary Fig. 10: Results of 3DVA showing different pattern of motions between 20S+monoUb-CyclinB-NT and 20S only structure. a, Central slices of the first three 3D variability components for the 20S only map. Positive (red) and negative (blue) values correspond to density to be added and subtracted from the mean density, respectively. **b**, Component 1 of 20S only map depicts a squashed motion of the whole complex. The dashed circles in the first two panels are identical perfect circles. It can be seen that the distances between the edge of the structure and the circle are different in the arrow-indicated direction (from blue to red map). **c,** Central slices of the first three 3D variability components for the

20S+monoUb-CyclinB1-NT map. Positive (red) and negative (blue) values correspond to density to be added and subtracted from the mean density, respectively. **d**, Component 1 of 20S+monoUb-CyclinB-NT exhibits dynamics in the gate region (indicated by an orange dashed circle) without an obvious overall shape change. Here, the red and blue maps are the two representative extreme maps. This rendering style is followed.

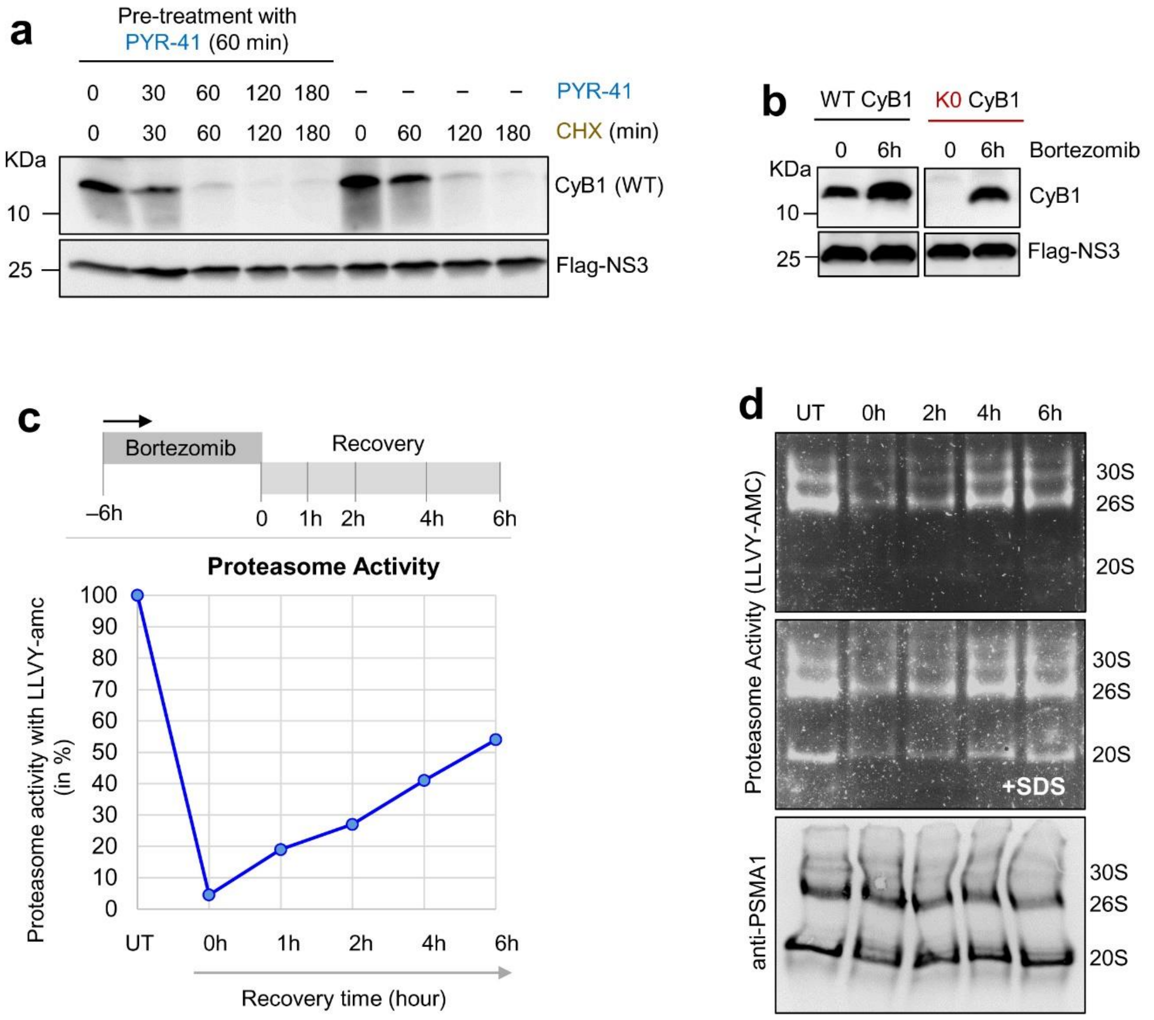

Supplementary Fig. 11: Effects of E1 or 20S inhibitors on CyclinB1 degradation a, Cycloheximide chase of CyclinB1 degradation. HA-CyclinB1-NT expressing HEK293T cells were treated with translation inhibitor cycloheximide (CHX; 50 µg/mL) and/or E1 ubiquitin activation enzyme inhibitor PYR-41 (10 µM) for the indicated time period followed by IB for Cyclin B1 (IB of NS3 serves as a control for cyclinB1 expression and as a gel loading control). **b**, Wild type (WT)-CyclinB1-NT or K0-CyclinB1-NT were transiently expressed in HEK293T cells with or without treatment of proteasome inhibitor bortezomib (1 µM) for 6 h. IB was performed using anti-CyclinB1-NT and anti-Flag antibodies. **c**, Bortezomib removal restores proteasome activity. HEK293T cells were pulse treated with bortezomib (0.5 µM) for 6 h. Then, bortezomib was removed and cells were grown in normal media for indicated time

periods. Native cell lysates were prepared at each time points and proteasome peptidase activity (LLVY-AMC) was measured. The graph shows the average proteasome peptidase activity of three technical repeats at each time-point. **d**, Native cell lysates from panel **c** were resolved by 4% native gel for proteasome in-gel proteolytic assay to evaluate proteasome activity or for transfer and immunoblotting of intact complexes using anti-PSMA1 antibody. Source data are provided as a Source Data file.

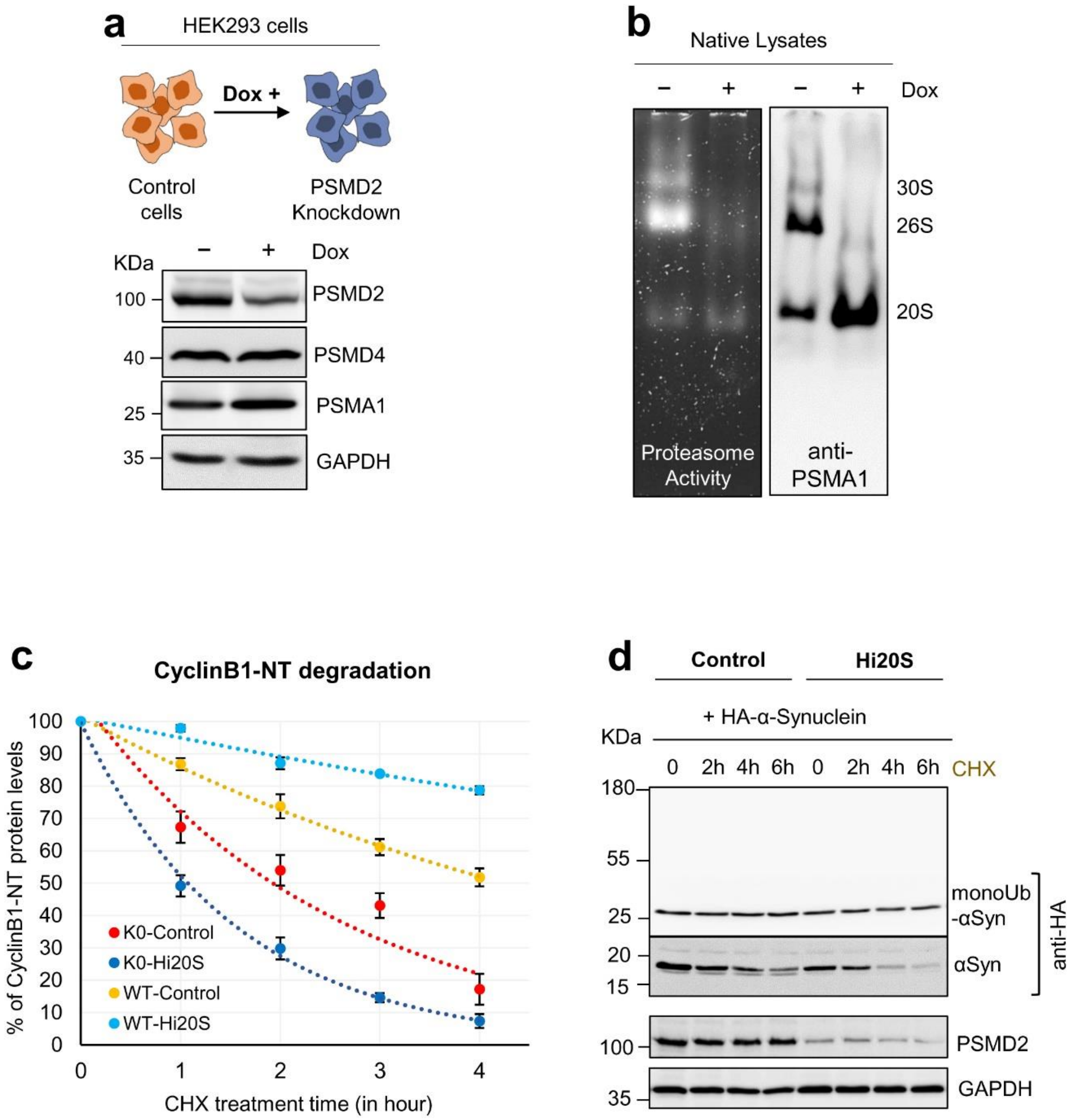

Supplementary Fig. 12: Efficient degradation of unstructured proteins in Hi20S cells generated by PSMD2 knockdown. a, Inducible PSMD2 knockdown (PSMD2-KD) HEK293T cell line was treated with doxycycline at 1 µg/mL for three passages to deplete PSMD2 protein levels. Immunoblot shows resulting changes to protein levels of representative proteasome subunits (PSMD4, PSMA1). The resulting cells are referred to herein as Hi20S cells. b, Hi20S cells or PSMD2-KD HEK293T cells grown without doxycycline as untreated

control were lysed with ATP-buffer and resolved by native gel to detect proteasome activity (left) or proteasome content (right). **c**, Control and Hi20S cells transiently expressing either HA-tagged WT-CyclinB1-NT or K0-CyclinB1-NT were pulsed with 0.5 µM bortezomib followed by cycloheximide chase. WT-Cyclin B1-NT and K0-Cyclin B1-NT protein levels were evaluated from anti-Cyclin B1 IB at each time-point of cycloheximide treatment. Average valaue of residual CyclinB1 and error bars (±SD) caclucated from three independent experiments. **d**, Stable inducible PSMD2-KD T-47D cells (grown with or without doxycycline; 1 µg/mL) transiently expressing HA-α-Synuclein were treated with cycloheximide (50 µg/mL) for up to 6 h. HA-α-Synuclein levels were detected by anti-HA immunoblotting. Source data are provided as a Source Data file.

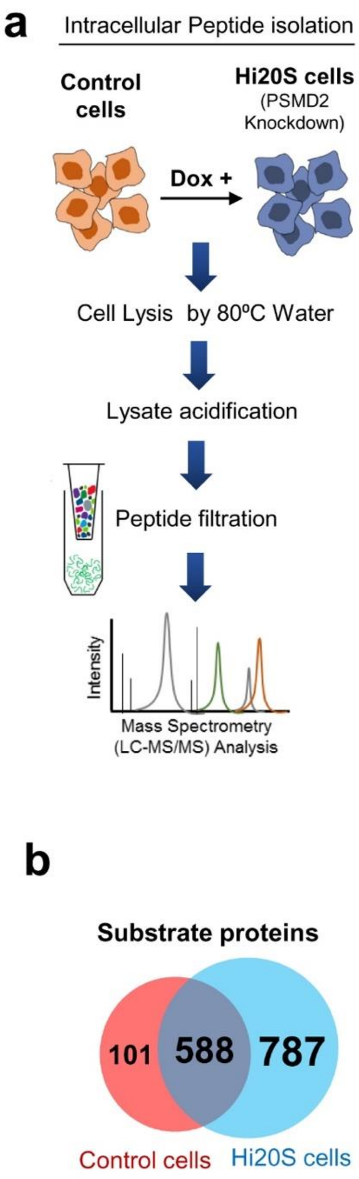

Supplementary Fig. 13: Intracellular peptidomics of WT and Hi20S cells. a, A method for intracellular peptide isolation and identification. Control and Hi20S HEK293T cells expressing HA-tagged K0 Cyclin B1-NT were grown with aminopeptidase inhibitor (1 μM CHR 2797) for 24 h. Intracellular peptides were captured as described in Method section and subject to non-tryptic LC-MS/MS analysis. **b**, Venn diagram represents potential proteasome substrates unique/common to each to each cell line (control, Hi20S). Proteins were assigned from intracellular peptides captured and scored based on intrinsic disordered elements.

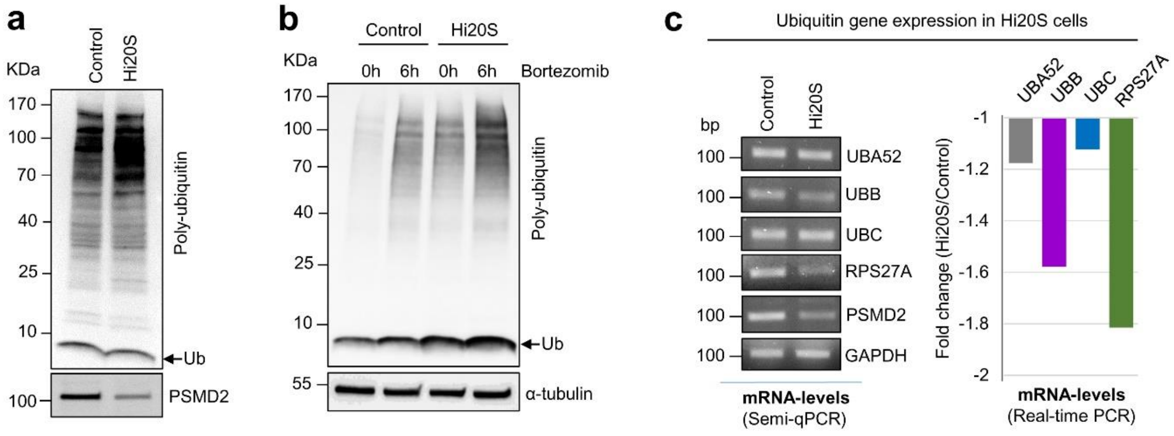

Supplementary Fig. 14: Ubiquitin landscape of control and Hi20S cells. Abundance of polyubiquitin conjugates and of free ubiquitin units in WT and Hi20S T-47D cells detected by IB using anti-ubiquitin antibody. **a**, basal levels, and **b**, upon 6h bortezomib treatment. **c**, RNA was isolated from control and Hi20S T-47D cells to detect levels of ubiquitin gene expression (expression of all four genes that encode for ubiquitin were evaluated). Relative mRNA levels of all four ubiquitin genes were represented in agarose gel image (left) by semi-qRT-PCR and in graph by Real-time PCR. GAPDH gene was used as the control for normalization (right). Source data are provided as a Source Data file.

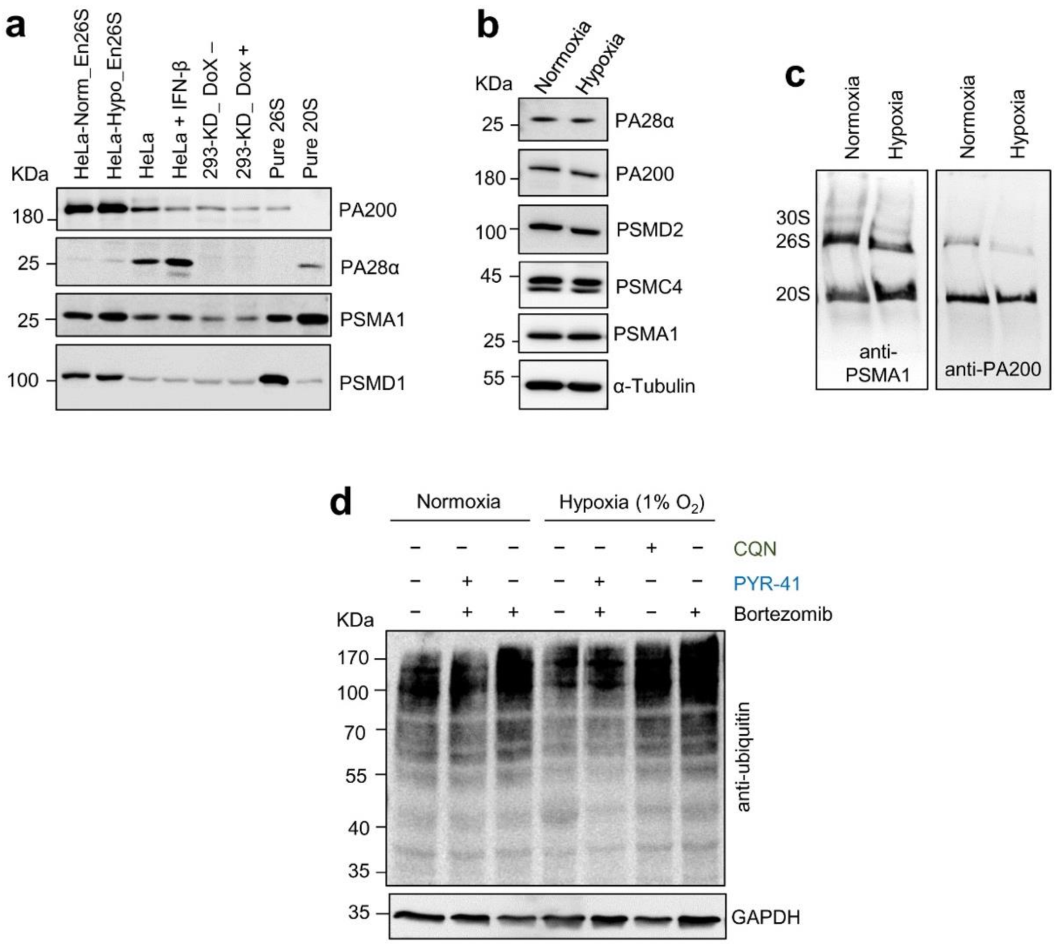

Supplementary Fig. 15: Hi20S conditions do not show significant association of alternative caps with 20S. a, Enriched proteasomes from HeLa cells grown under normoxia or hypoxia (1% O_2) for 24 h, HeLa cell lysate treated with interferon beta (INFβ), Cell Lysates from Hi20S HEK293 cells, orpurified 26S/20S proteasomes were resolved by SDS-PAGE and immunoblotted with indicated antibodies. **b**, HeLa cells were grown either under normoxia or hypoxia (1% O_2) for 24 h. Native cell lysates were resolved in 4% native gel for native immunoblot using anti-PA200 (PSME4) antibody. **c**, HeLa cells were grown either under normoxia or hypoxia (1% O_2) for 24 h. Cell lysates were resolved in SDS-PAGE for immunoblot to detect PA200 (PSME4) and PA28α (PSME1). **d**, Polyubiquitin content of HeLa cells grown under normoxia or under hypoxia either treated with chloroquine, PYR-41 or bortezomib for 6h as indicated. IB using anti-ubiquitin antibody. Source data are provided as a Source Data file.

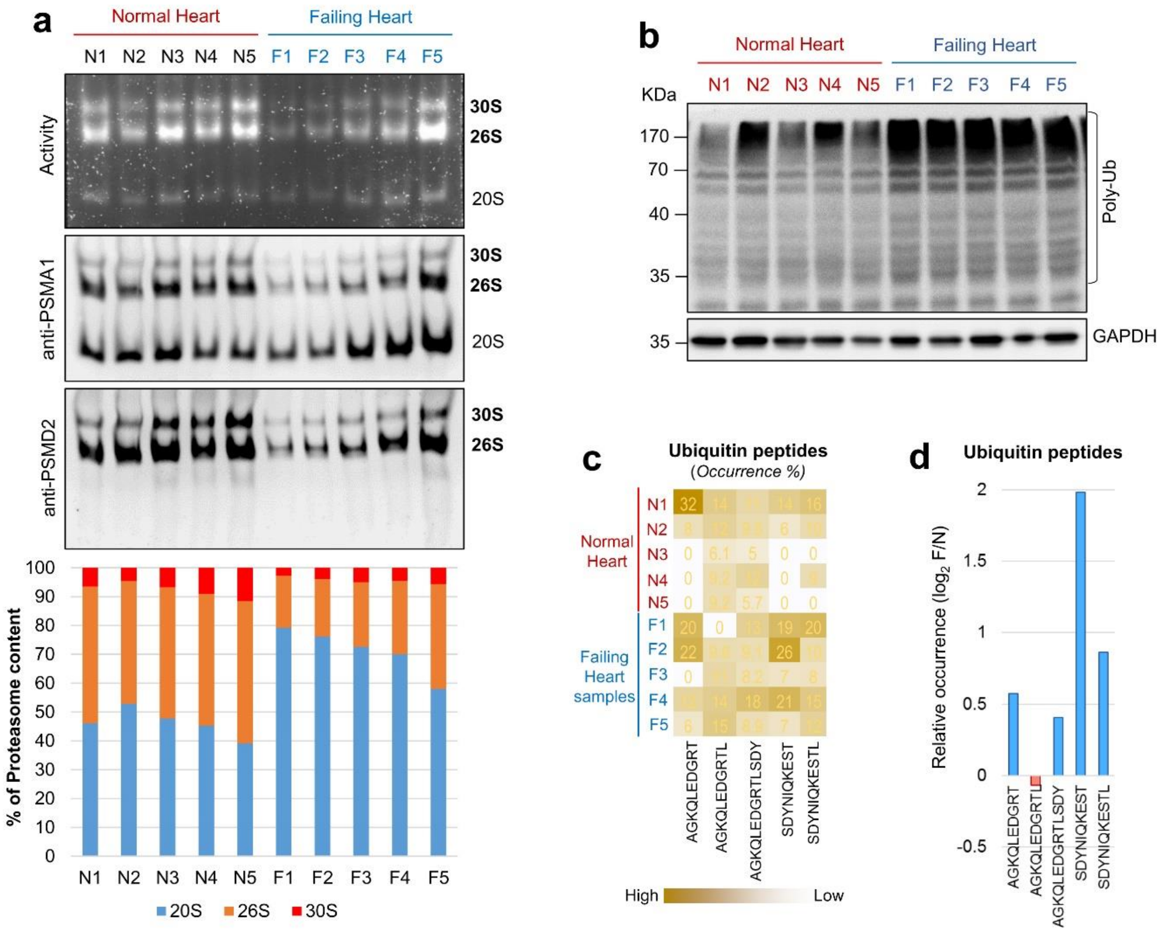

Supplementary Fig. 16: Failing heart displayes elevated levels of 20S complexes, of polyubiquitin conjugates, and of intracellular ubiquitin peptides. a, Proteasome activity (upper) and protein content (native IB: middle) of human heart muscle tissue under Failing (N = 5) or Normal (N = 5) conditions. Proteasome enrichment from frozen tissue followed the protocol in Methods section and ratio of proteasome species for each sample was measured from anti-PSMA1 probed native IB. The bar graph (lower) represents the percentage of proteasome content of Normal heart samples (N = 5) and Failing heart samples (N = 5). **b**, Polyubiquitin content of human heart muscle lysates IB using antiUb. **c**, Heat map represents the ubiquitin-derived peptides in heart muscle tissues. Human frozen heart muscle tissue under Failing (N = 5) and Normal (N = 5) conditions were subjected to intracellular peptide isolation following the protocol in Methods section and quantitatively analyzed for ubiquitin peptides

by LC-MS/MS. The values represent the relative occurrence of each ubiquitin peptide across 10 samples calculated from their LFQ-intensity. **d**, The bar graph represents the relative occurrence of each ubiquitin peptides (calculated from integrated LFQ intensity) in Failing heart samples compared to normal heart samples. Source data are provided as a Source Data file.

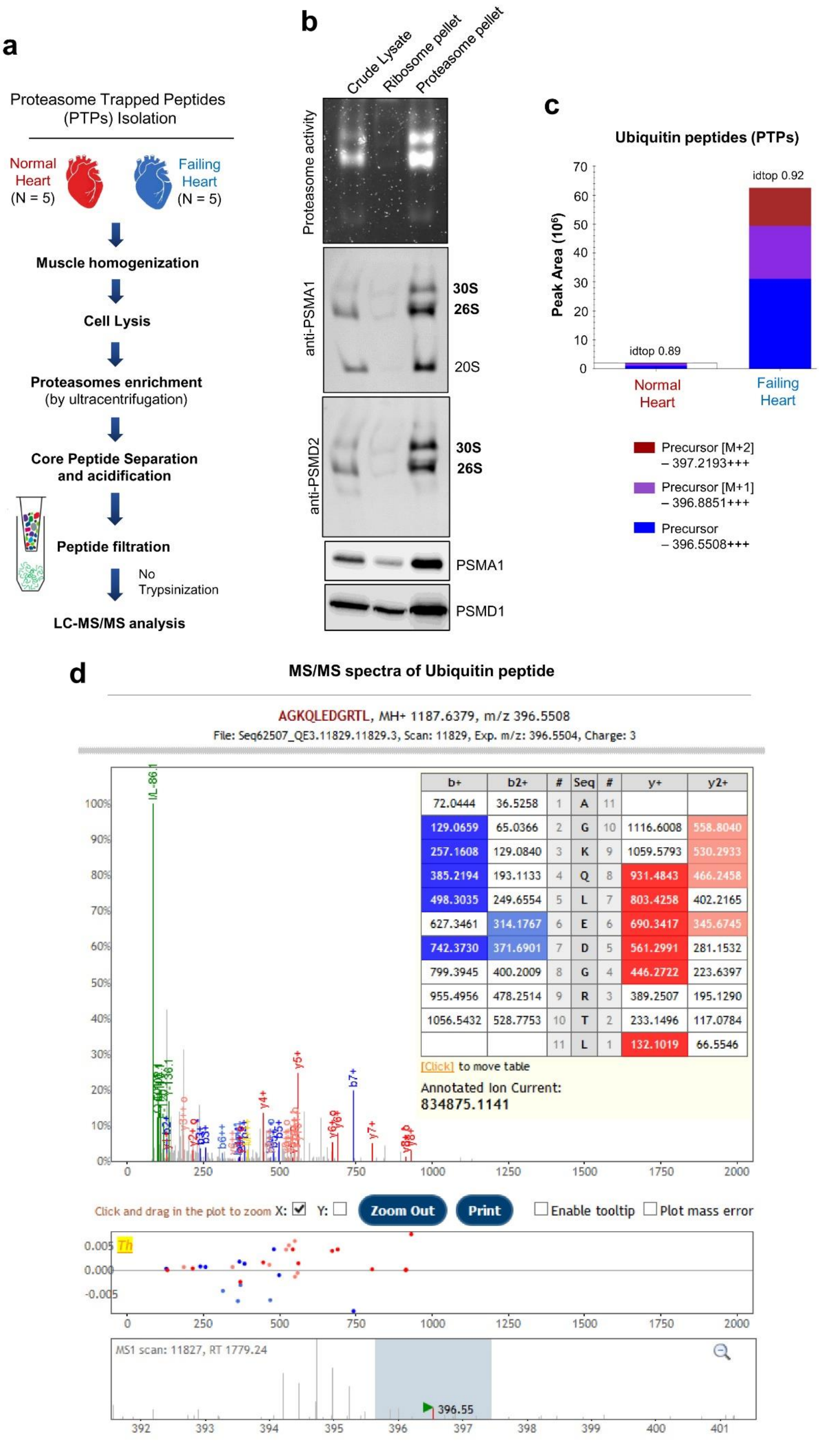

b+	b2+	#	Seq	#	y+	y2+
72.0444	36.5258	1	A	11		
129.0659	65.0366	2	G	10	1116.6008	558.8040
257.1608	129.0840	3	K	9	1059.5793	530.2933
385.2194	193.1133	4	Q	8	931.4843	466.2458
498.3035	249.6554	5	L	7	803.4258	402.2165
627.3461	314.1767	6	E	6	690.3417	345.6745
742.3730	371.6901	7	D	5	561.2991	281.1532
799.3945	400.2009	8	G	4	446.2722	223.6397
955.4956	478.2514	9	R	3	389.2507	195.1290
1056.5432	528.7753	10	T	2	233.1496	117.0784
		11	L	1	132.1019	66.5546

Supplementary Fig. 17: Failing heart shows elevated levels of ubiquitin peptides associated with proteasomes. **a**, Scheme describes approach for Proteasomal Trapped Peptides (PTPs) isolation and MS/MS analysis from human heart muscle tissues. **b**, Qualitative analysis of proteasome enrichment method. Each fraction during the process (see Methods section); e.g., crude lysates, ribosomal pellet and proteasome pellet were resolved in native gel to detect proteasome activity (upper) or proteasome protein content by native IB (middle). Lower denature PAGE IB shows two proteasome subunit levels. **c**, Intensity of Ubiquitin peptides associated with enriched proteasome from heart muscle tissues. Proteasome was isolated from frozen human heart tissue, and integrated intensity proteasome trapped peptides (PTPs) were calculated by MS/MS. Bar graph summarizes ubiquitin peptides (from all charged forms) in all samples from both conditions. **d**, The MS/MS spectra of the ubiquitin peptide identified. Source data are provided as a Source Data file.

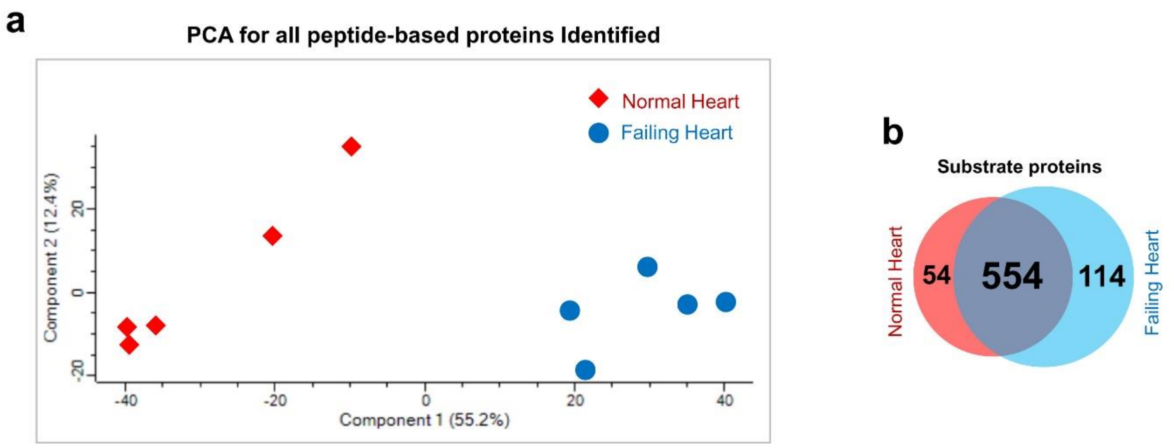

Supplementary Fig. 18: Failing heart shows peptides from proteins with higher disordered score. a, The graph shows the principal component analysis (PCA) of MS/MS detected all intracellular peptide-based proteins of failing and non-failing heart tissue samples (N=10). **b**, Venn diagram represents potential proteasome substrates unique/common to heart sample set. Proteins were assigned from intracellular peptide captured and scored based on intrinsic disordered elements.

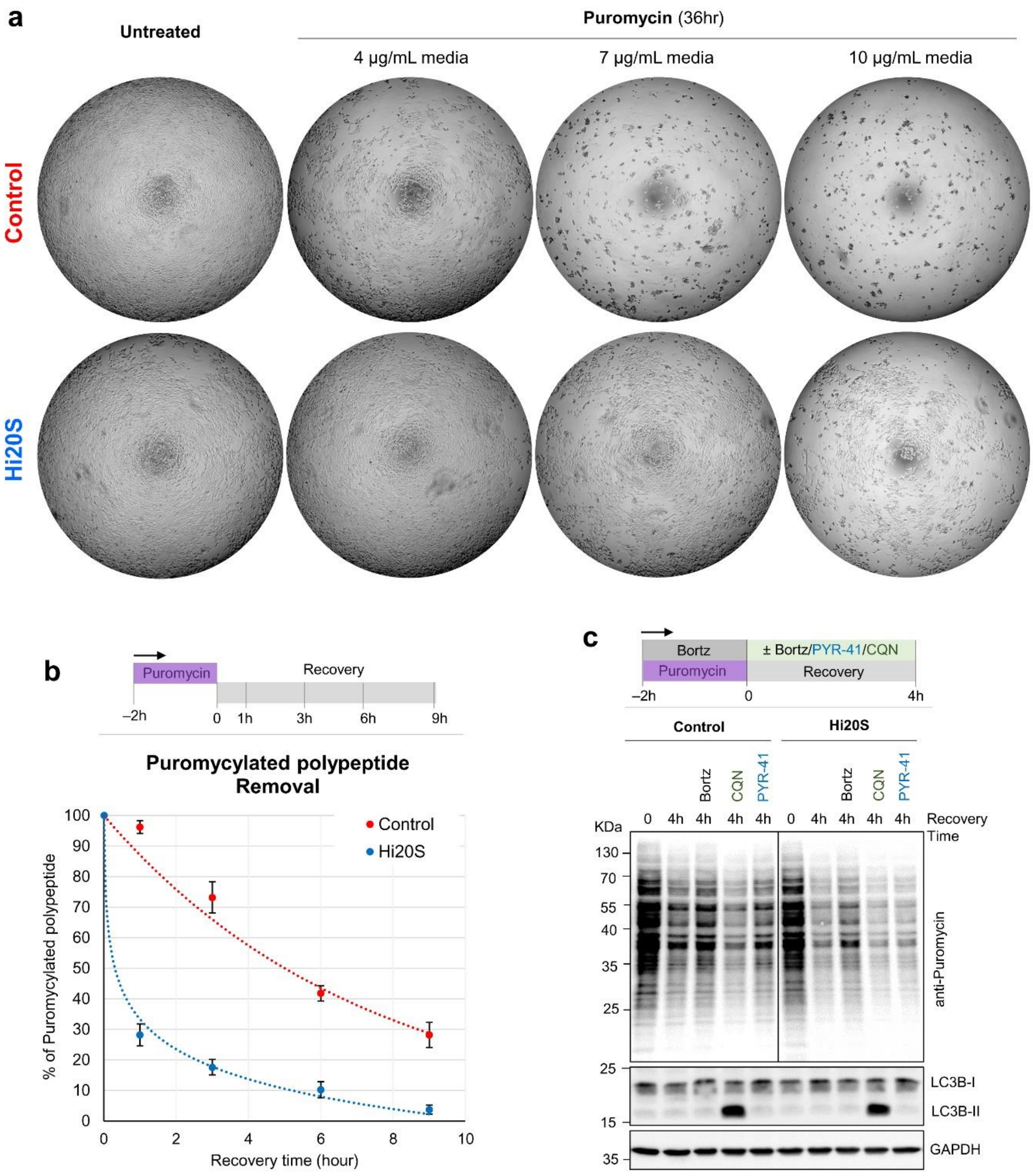

Supplementary Fig. 19: Hi20S cells survive higher concentrations of puromycin by efficiently remove puromycylated nascent polypeptides. a, Phase contrast images of culture plates showing survival of control and Hi20S cells after 36 h of puromycin treatment at increasing concentration. **b**, Control and Hi20S HEK293 cells were given a pulse of puromycin (5 µg/mL) for 2 h. Followed by a recovery for different time periods as indicated and IB was performed to detect puromycylated polypeptide susing anti-puromycin antibody. Graph shows

the rate of puromycylated polypeptides removal. Data represents the average percentage of puromycylated polypeptides (±SD error bar) at each time points of the recovery phase, quantified from IB of three independent experiments. **c**, Control and Hi20S HEK293T cells were given a pulse of puromycin (5 μg/mL) for 2 h. Followed by a recovery for different time periods with/without bortezomib (1 μM)/chloroquine (50 μM)/ PYR-41 (10 μM) as indicated and IB was performed. Source data are provided as a Source Data file.

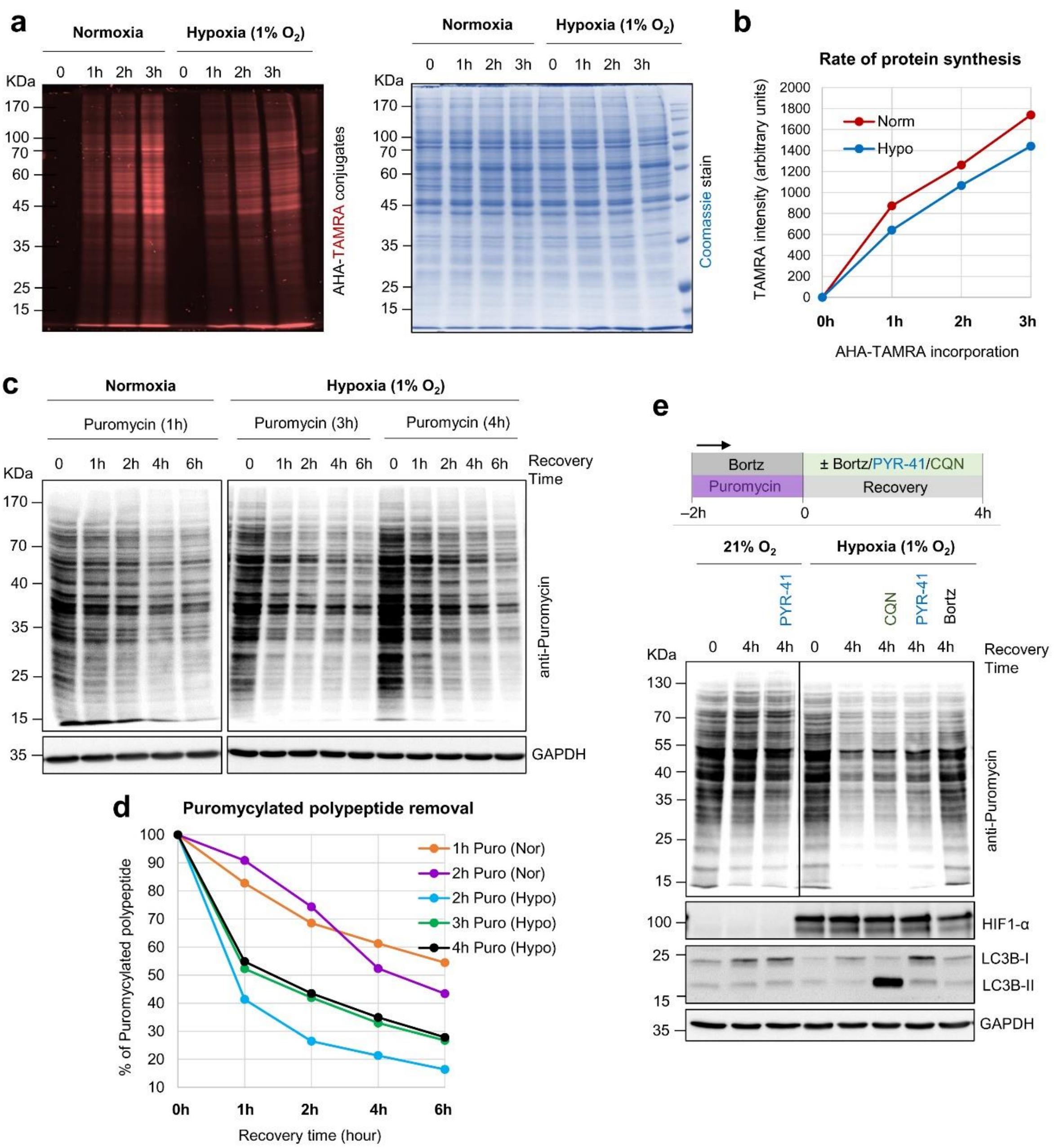

Supplementary Fig. 20: Puromycylated polypeptides are cleared out efficiently by proteasome under hypoxia. a, HeLa cells were grown under normoxia or hypoxia (1% O_2) for 24 h followed by pulse-treatment with 0.2mM L-azidohomoalanine (AHA) for indicated time periods. The cells lysates were treated with TAMRA-alkyne and were resolved by SDS-PAGE to detect AHA-TAMRA conjugated nascent protein under 580nm. The Coomassie stained gel indicated the protein loading control. **b**, Graph represents the average rate of protein synthesis under normoxia or hypoxia (1% O_2) calculated from AHA-TAMRA conjugated

nascent protein of two experimental repeat. **c**, HeLa cells were grown under hypoxia for 24 h followed by puromycin (5 µg/mL) treatment for different time periods and then recovered after removal of puromycin (Recovery) over 6 h. Cell lysates were resolved by SDS-PAGE to detect puromycylated polypeptides by IB. **d**, Graph represents the rate of puromycin-conjugate removal under normoxia or hypoxia calculated from anti-puromycin IB. Puromycin (5 µg/mL) treatment was given to cells for different time periods as indicated. **e**, HeLa cells were grown under either normoxia or hypoxia for 24 h followed by 2 h puromycin (5 µg/mL), pulse with/without bortezomib (1 µM)/PYR-41 (10 µM)/Chloroquine (50 µM) and then recovery for indicated time periods with/without the inhibitors as indicated. Cell lysates were resolved in SDS-PAGE to detect puromycylated polypeptides by IB. Source data are provided as a Source Data file.

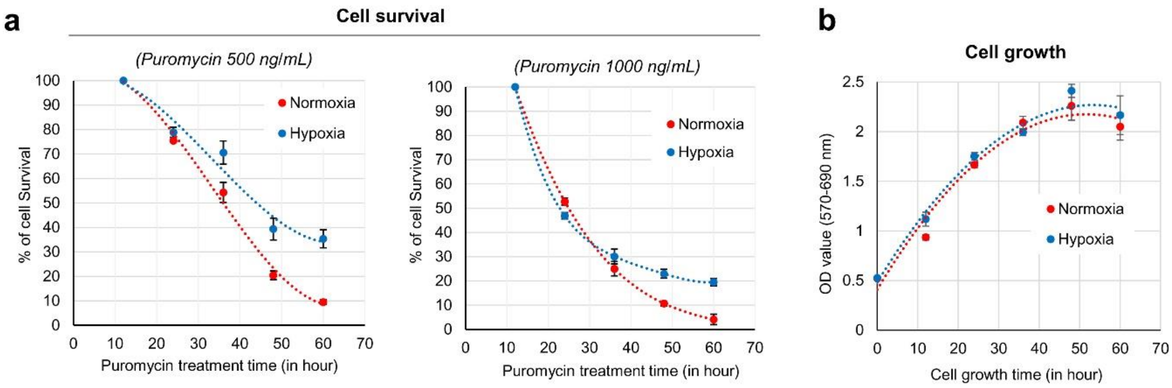

Supplementary Fig. 21: HeLa cells survive exposure to higher levels of puromycin under hypoxia. a, The graphs represent cell survival upon puromycin exposure during hypoxia. HeLa cells were grown under normoxia or hypoxia (1% O_2) with different puromycin concentration (500 ng/mL and 1000 ng/mL) for up to 60 h. Cell survival was quantified by MTT assay. Data represents the average of three experimental values (±SD error bar). **b**, Growth curve of HeLa cells growing under normoxia or hypoxia. Cell growth was calculated from MTT assay. Data represents the average of three experimental values (±SD error bar). Source data are provided as a Source Data file.

Supplementary Table 1. Branched peptide detected around the isopeptide bond between proximal ubiquitin and CyclinB1.

Probability	Branched Peptide detected
1	G<br>G<br>RLPLRKEARPSATGR
1	G<br>G<br>PLRKEARPSATGRVID
1	G<br>G<br>RLPLRKEARPSATGRVI
1	G<br>G<br>QARLPLRKEARPSATGRVI
0.9921	R<br>G<br>G<br>ARLPLRKE
0.9801	R<br>G<br>G<br>ARLPLRKEA
0.9936	R<br>G<br>G<br>LPLRKEARPSATGRVI
0.9847	R<br>G<br>G<br>PLRKEARPSA
1	R<br>G<br>G<br>PLRKEARPSATGR
1	R<br>G<br>G<br>PLRKEARPSATGRVIDR
0.9787	R<br>G<br>G<br>QARLPLRKE
0.9941	R<br>G<br>G<br>QARLPLRKEARPSATGRVID
0.9918	R<br>G<br>G<br>RLPLRKEARPSA
1	R<br>G<br>G<br>RLPLRKEARPSATGR
0.9783	R<br>G<br>G<br>RLPLRKEARPSATGRVI
1	R<br>G<br>G<br>RLPLRKEARPSATGRVIDRRLPRPLE
0.9938	L<br>R<br>G<br>G<br>PLRKEARPSATGR
0.9968	L<br>R<br>G<br>G<br>PLRKEARPSATGRVI

Supplementary Table 2. Real time PCR primers.

**Gene name**	**Sequence**
GAPDH	Fw-5'TGCACCACCAACTGCTTAG3'<br>Rv-5'GATGCAGGGATGATGTTC3'
PSMD2	Fw-5'ATGCTGGTTACGTTTGATGAGGAG3'<br>Rv-5'GCGTCTGGAACCCTGTGATAGTC3'
UBC	Fw-5'GTGTCTAAGTTTCCCCTTTTAAGG3'<br>Rv-5'TTGGGAATGCAACAACTTTATTG3'
UBB	Fw-5'CTTTGTTGGGTGAGCTTGTTTGT3'<br>Rv-5'GACCTGTTAGCGGATACCAGGAT3'
UBA52	Fw-5'CTGCGAGGTGGCATTATTGAG3'<br>Rv-5'GTTGACAGCACGAGGGTGAAG3'
RPS27A	Fw-5'TCGTGGTGGTGCTAAGAAAAGG3'<br>Rv-5'TTCAGGACAGCCAGCTTAACCT3'

Supplementary Table 3. Cryo-EM data collection and refinement statistics

	20S alone	20S+monoUb-CyclinB1-NT		
		S0	S1	S2
**Data collection**				
EM equipment	Titan Krios		Titan Krios	
Voltage (kV)	300		300	
Detector	K2 Summit		K2 Summit	
Pixel size (Å)	1.318		1.318	
Electron dose (e^{-}/Å^{2})	38		38	
Exposure time (s)	7.6		7.6	
Frames	38		38	
Defocus range (µm)	-0.9 to -1.8		-0.9 to -1.8	
**Reconstruction**				
Software	Relion 3.0		Relion 3.0	
Raw micrographs	748		3,125	
Final particles	154,436	282,834	72,086	179,590
Final resolution (Å)	3.22	3.38	4.47	3.88
**Atomic modeling**				
Software	Phenix & COOT		Rosetta, Phenix & COOT	Phenix & COOT
Ramachandran favored	96.91%		93.10%	96.87%
Ramachandran allowed	3.06%		6.77%	3.09%
Ramachandran outliers	0.03%		0.13%	0.03%
Rotamer outliers	0.00%		0.06%	0.00%
C-beta deviations	0		0	0
Clashscore	7.89		6.64	9.07
Rms deviations				
Bond length (Å)	0.0041		0.0035	0.0039
Bond Angle (°)	0.97		0.89	0.95
MolProbility score	1.62		1.81	1.67

Supplementary Table 4. The search parameters for ubiquitin remnants by MSFragger

Search number	Ub Remnant	Precursor tolerance	Fragment mass tolerance	Digestion	Variable modifications
1	none	20ppm	25ppm	Non-specific	(+15.9949) M<br>(+42.02) Protein N-term
2	G	20ppm	25ppm	Non-specific	(+15.9949) M<br>(+42.02) Protein N-term<br>(+ 57.021460) K
3	GG	20ppm	25ppm	Non-specific	(+15.9949) M<br>(+42.02) Protein N-term<br>(+114.04292) K
4	GGR	20ppm	25ppm	Non-specific	(+15.9949) M<br>(+42.02) Protein N-term<br>(+270.144039) K
5	GGRL	20ppm	25ppm	Non-specific	(+15.9949) M<br>(+42.02) Protein N-term<br>(+383.228103) K
6	GGRLR	20ppm	25ppm	Non-specific	(+15.9949) M<br>(+42.02) Protein N-term<br>(+539.329214) K

Supplementary Methods

Chemical synthesis and ubiquitination of CyclinB1 substrates

HA-CyclinB1-NT Sequence:

YPYDVPDYANleALRVTRNSRINAENRARINNleAGARRVPTAPAATSRPGLRPRT**A**L GDIGNRVSEQLQARNlePLRK**(64)**EARPSATGRVIDRRLPRPLERVPNle

(YPYDVPDYA = HA tag; Nle = Norleucine replacing methionine in CyclinB1 sequence).

For synthesis of HA-CyclinB1-NT, the sequence was divided into two fragments:

1. **C**LGDIGNRVSEQLQARNlePMR**K(64)**EARPSATGRVIDRRLPRPLERVPNle
2. YPYDVPDYANleALRVTRNSRINAENRARINNleAGARRVPTAPAATSRPGLRPRT-Nbz

Synthesis of fragment 1, CyclinB1-NT (53-97):

CLGDIGNRVSEQLQARNlePMR**K(64)**EARPSATGRVIDRRLPRPLERVPNle

The synthesis was carried out using Fmoc-SPPS on Rink amide resin (0.27 mmol/g, 0.1 mmol scale). Peptide synthesis was performed on peptide synthesizer in presence of 0.4 mmol of amino acid, HCTU) and 0.8 mmol of DIEA. For the synthesis of fragment **1**, the pre-swollen resin was treated with 20% piperidine in DMF containing 0.1 mmol HOBt (3 ×3 cycles) to remove the Fmoc-protecting group. The Thz protected δ-mercaptolysine was manually coupled for 1.5 h at the position Lysine64 using HATU/DIEA. The remaining amino acids were coupled on an automated peptide synthesizer.

Cleavage from the resin: The resin was washed with DMF, MeOH, DCM and dried. The peptide was cleaved using TFA:triisopropylsilane (TIS):water (95:2.5:2.5) cocktail for 2 h. The cleavage mixture was filtered and the combined filtrate was added drop-wise to a 10 fold volume of cold ether and centrifuged. The precipitated crude peptide was dissolved in acetonitrile-water (1:1) and was further diluted to ~30% with water and lyophilized. The HPLC analysis was carried out on a C18 analytical column using a gradient of 0-60% B over 30 min. For preparative HPLC, the same gradient was used to purify the fragment **1** in ~50% yield.

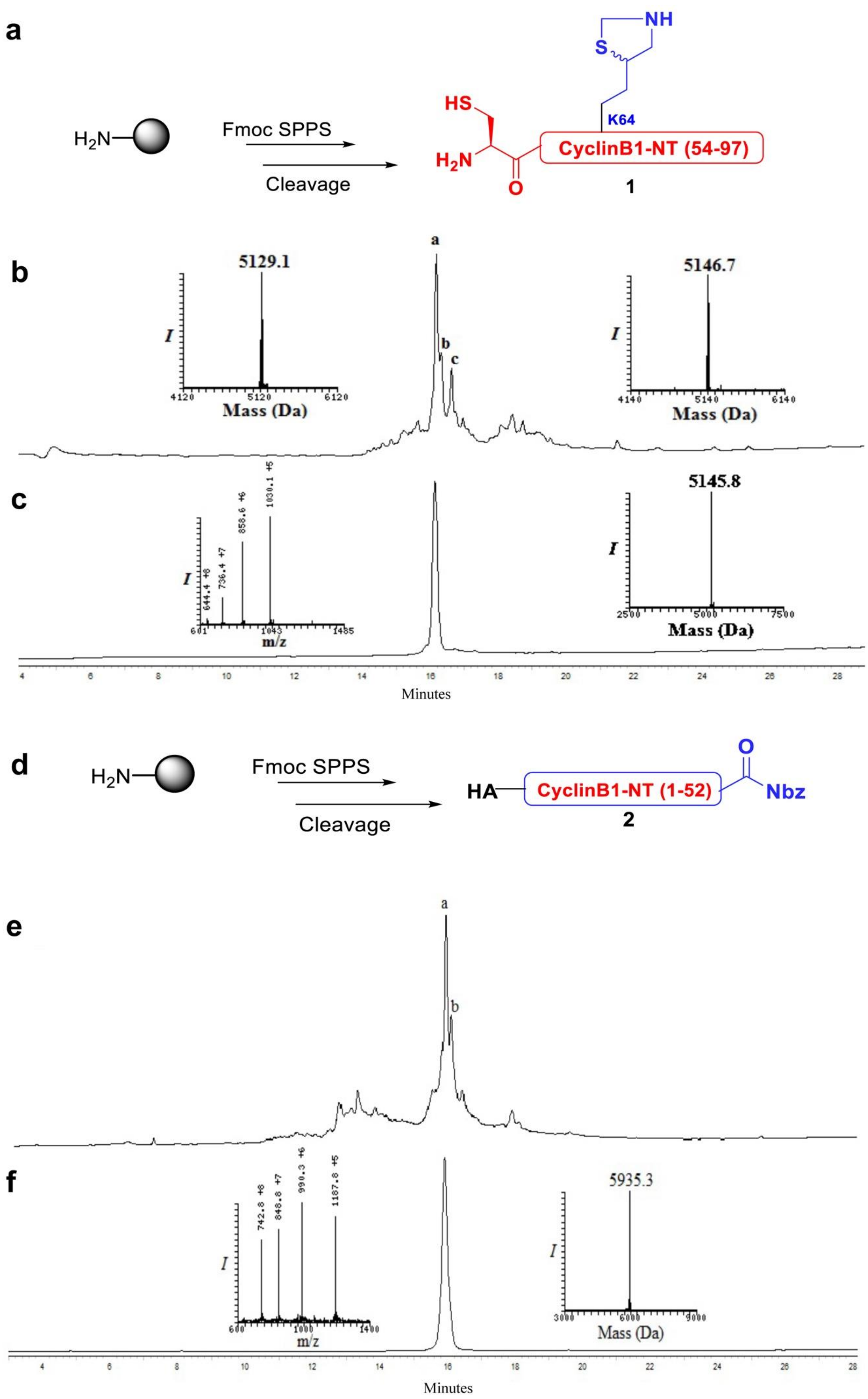
a
H2N
Fmoc SPPS
Cleavage
NH
S
HS
K64
H2N
O
CyclinB1-NT (54-97)
1
b
5129.1
I
4120
5120
6120
Mass (Da)
a
b
c
5146.7
I
4140
5140
6140
Mass (Da)
c
1030.1 +5
858.6 +6
736.4 +7
644.4 +8
I
601
1043
1485
m/z
5145.8
I
2500
5000
7500
Mass (Da)
4
6
8
10
12
14
16
18
20
22
24
26
28
Minutes
d
H2N
Fmoc SPPS
Cleavage
O
HA
CyclinB1-NT (1-52)
Nbz
2
e
a
b
f
742.8 +8
848.8 +7
990.3 +6
1187.8 +5
I
600
1000
1400
m/z
5935.3
I
3000
6000
9000
Mass (Da)
4
6
8
10
12
14
16
18
20
22
24
26
28
Minutes

Supplementary Fig. 22: Synthesis of individual HA-CyclinB1 fragments. **a**, Scheme depicts synthesis of Fragment **1.** **b,** Analytical HPLC and mass traces of crude fragment **1**, **CyclinB1-NT (53-97)**. Peak *a* corresponds to fragment **1** with the observed mass 5146.7 Da (calculated 5146.9 Da); Peak *b* corresponds to a deletion -18 from the parent peptide. **c**, Analytical HPLC and mass traces of purified fragment **1**. **d**, Scheme depicts synthesis of Fragment **2**, (Nbz = N-acyl-benzimidazolinones). **e**, Analytical HPLC and mass traces of crude fragment **2**, **HA-CyclinB1-NT (1-52)-Nbz**. Peak *a* corresponds to fragment **2** with the observed mass 5935.3 Da (calculated 5935.5 Da); Peak *b* corresponds to a deletion from the parent peptide. **f**, Analytical HPLC and mass traces of purified fragment **1**.

Synthesis of fragment 2, HA-CyclinB1-NT (1-52)-Nbz

YPYDVPDYA NleALRVTRNSRINAENRARINNleAGARRVPTAPAATSRPGLRPRT-Nbz

The synthesis was carried out using the N-acylurea method on Rink amide resin (0.27 mmol/g, 0.1 mmol scale). Mono-Fmoc-3,4-diaminobenzoic acid (Fmoc-Dbz) activated with HBTU/HOBt was coupled to the resin for 1 h (2 cycles). Subsequently, the resin was washed with DMF and DCM (3 x 5 mL) and was treated with a solution of allyl chloroformate (50 equivalents) and DIEA (0.2 mmol) in DCM for 12 h. Following the Fmoc removal Peptide synthesis was performed on peptide synthesizer in presence of 0.4 mmol of amino acid and coupling agent (HCTU) while 0.8 mmol of DIEA. The remaining amino acids were coupled using peptide synthesizer as described above. After completion of peptide synthesis, The Alloc-protected resin was washed and swollen in DCM. A solution of $PhSiH_3$ 20 equivalents with respect to initial loading of the resin and Pd (PPh3) 4 (0.35 eq) in DCM was added to the resin and shaken for 1 h at 25^0C to remove alloc protection. The resin was washed with DCM and a solution of *p*-nitrophenylchloroformate (100 mg, 5 equivalents) in 5 mL of DCM was added and shaken for 1 h at RT. The resin was washed with DCM (3 × 5 mL). Following these steps, a solution of 0.5 M DIEA in DMF (5 mL) was added and shaken for additional 30 min to complete the cyclization. The resin was washed using DMF (3 × 5 mL). Cleavage and purification were carried out as described above to afford the fragment **2** in 40-50% yield.

Native chemical ligation between fragment 1, CyclinB1-NT (53-97) and fragment 2, HA-CyclinB1-NT (1-52)-Nbz:

Ligation: fragment **1**, CyclinB1-NT (53-97) (10 mg, 1.9 × 10^{-3} mmol) and fragment **2**, HA-CyclinB1-NT (1-52)-Nbz (13.8 mg, 2.3 × 10^{-3} mmol) were dissolved in argon purged 6 M Gn·HCl, 200 mM Na_2HPO_4 buffer (971 µL, 2 mM) containing 20 equivalents of MPAA and 10 equivalents of TCEP, pH ~7.3. The reaction was incubated at 37°C for 4 h. Progress of the reaction was monitored by analytical HPLC using C18 column with a gradient of 5-55% buffer B over 45 min. After completion of ligation, the reaction mixture was diluted to 1mM concentration with a solution of 30 equivalents of methoxylamine and 15 equivalents of TCEP at pH = 4. The reaction mixture was incubated in 37ºC for 12 h. The progress of the reaction was monitored by analytical HPLC using C18 column with a gradient of 5-55% B over 45 min and LC-MS analysis. For semi-preparative HPLC, the same gradient was used to isolate the ligation product **3** in 63% yield (~13 mg).

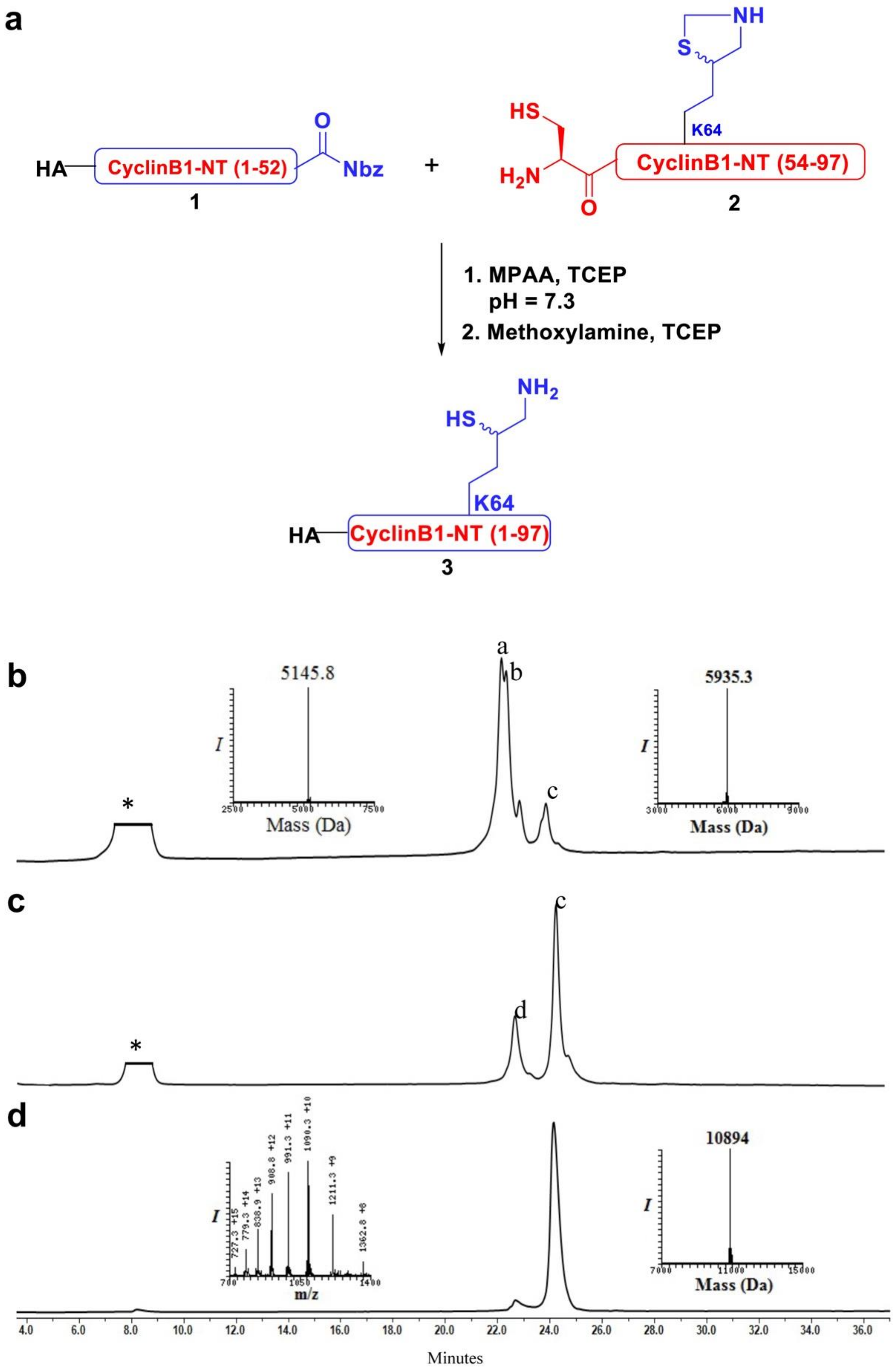

Supplementary Fig. 23: Synthesis of HA-CyclinB1-NT. **a**, Scheme depicts synthesis of HA-CyclinB1-NT (1-97), **3**. **b**, Analytical HPLC and mass traces for the ligation of fragment **1** and fragment **2**. Ligation at time zero, peak *a* corresponds to fragment **1**, peak *b* corresponds to

fragment **2**, peak ***c*** corresponds to ligation product **3**. **c**, Ligation after 6 h, peak *c* corresponds to ligation product **3** with the observed mass 10894 Da (calculated 10893 Da), peak *d* corresponds to Nbz hydrolyzed fragment **2**. **d**, Analytical HPLC and mass traces purified HA-CyclinB1-NT, **3**.

Desulfurization of HA-CyclinB1-NT

3 was subjected for the desulfurization using VA-044 (20 equivalents, 0.1 M), TCEP (0.25 M) in the presence of 10% t-BuSH (v/v) at 37^{0}C for 6 h. The reaction was followed using analytical HPLC (C18 column) and a gradient of 5-55% B over 60 min and LC-MS analysis. For semi-preparative HPLC, the same gradient was used to afford the desulfurized HA-CyclinB1-NT ~60% yield.

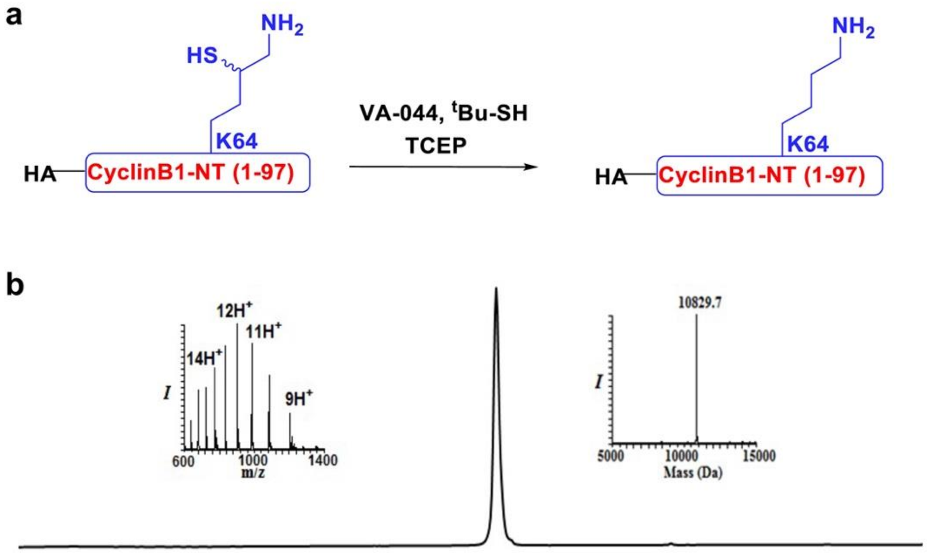

Supplementary Fig. 24: a, Scheme depicts Desulfurization of HA-CyclinB1-NT-(1-97*)*. **b**, Analytical HPLC and mass traces for the desulfurized and purified HA-CyclinB1-NT with the observed mass 10829.7 Da (calculated 10829.5 Da)

Synthesis of Ubiquitin Monomers

The synthesis of Myc-Ub1-N-Methyl-Cysteine, Ub2/3(K48*)-N-Methyl-Cysteine and Flag-Ub4-N-Me-Cysteine were carried on Rink amide resin (0.27 mmol/g, 0.1 mmol scale). The pre-swollen resin was treated with 20% piperidine in DMF containing 0.1 mmol HOBt (3 cycle 3 min each) to remove resin bound Fmoc-protecting group. Initially, Fmoc-S-*o*-nitrobenzyl N-methyl cysteine activated with HATU/DIEA was coupled to the resin for 1.5 h. Subsequently, the resin was washed with DMF. Following the Fmoc removal, all amino acids were coupled using an automated peptide synthesizer in presence of 0.4 mmol of amino acid, 0.8 mmol of DIEA and 0.4 mmol of coupling agent (HCTU) to the initial loading of the resin. Pseudoproline dipeptides Leu-Ser , Asp-Gly(Dmb), Ile-Thr and Leu-Thr were manually coupled at positions Leu56-Ser57 , Asp52-Gly53, Ile13-Thr14 and Leu8-Thr9 respectively using 0.25 mmol of Fmoc-Leu-Ser(ψMe, Mepro)-OH, Fmoc-Asp(OtBu)-(Dmb)Gly-OH, Fmoc-Ile-Thr(ψMe, Mepro)-OH and Fmoc-Leu-Thr(ψMe, Mepro)-OH respectively. During synthesis of Myc-Ub1-N-Me-Cysteine, Ub2/3(K48*)-N-Me-Cysteine 0.15 mmol thiazolidine (Thz)-protected δ-mercaptolysine was manually coupled for 2 h at the position 48 using HATU/DIEA. Analytical cleavage and HPLC analysis were performed after these couplings to ensure complete reaction. Norleucine was used in place of Met1 in ubiquitin sequence to avoid oxidation during synthesis. After the completion of synthesis, ubiquitin analogues were cleaved from resin as follow.

Cleavage from resin: The resin was washed with DMF, DCM and dried over high vacuum. A cocktail of TFA:DCM:triisopropylsilane:water (90:5:2.5:2.5) was added to resin and reaction mixture was shaken for 2 h at RT. The resin was filtered, and the combined filtrate was added dropwise to a 10 fold volume of cold ether and centrifuged. The precipitated crude peptide was dissolved (~70%) in acetonitrilewater (1:1) and was further diluted to ~30% with water and lyophilized. Myc tag- EQKLISEEDL and Flag tag- DYKDDDDK

Thz deprotection of Myc-Ub1K48

The crude peptide (50 mg) was dissolved in 6M Gn·HCl buffer to a final concentration of ~3 mM. Hydrazine hydrate (80% solution, 100 equivalents) was added and kept at 25^{0}C for 1 h (pH ~7). To the above reaction mixture, methoxylamine (0.2 M, 15 equivalents) and tris-(2-carboxyethyl)-phosphine (TCEP, 30 equivalents) were added and incubated at 37^{0}C for 3 h to unmask the δ-mercaptolysine completely. The reaction was monitored using an analytical HPLC (C18 column) and a gradient of 0-60% B over 30 min and LC-MS analysis. For HPLC purification, a similar gradient was used to afford the corresponding peptide in ~7% yield.

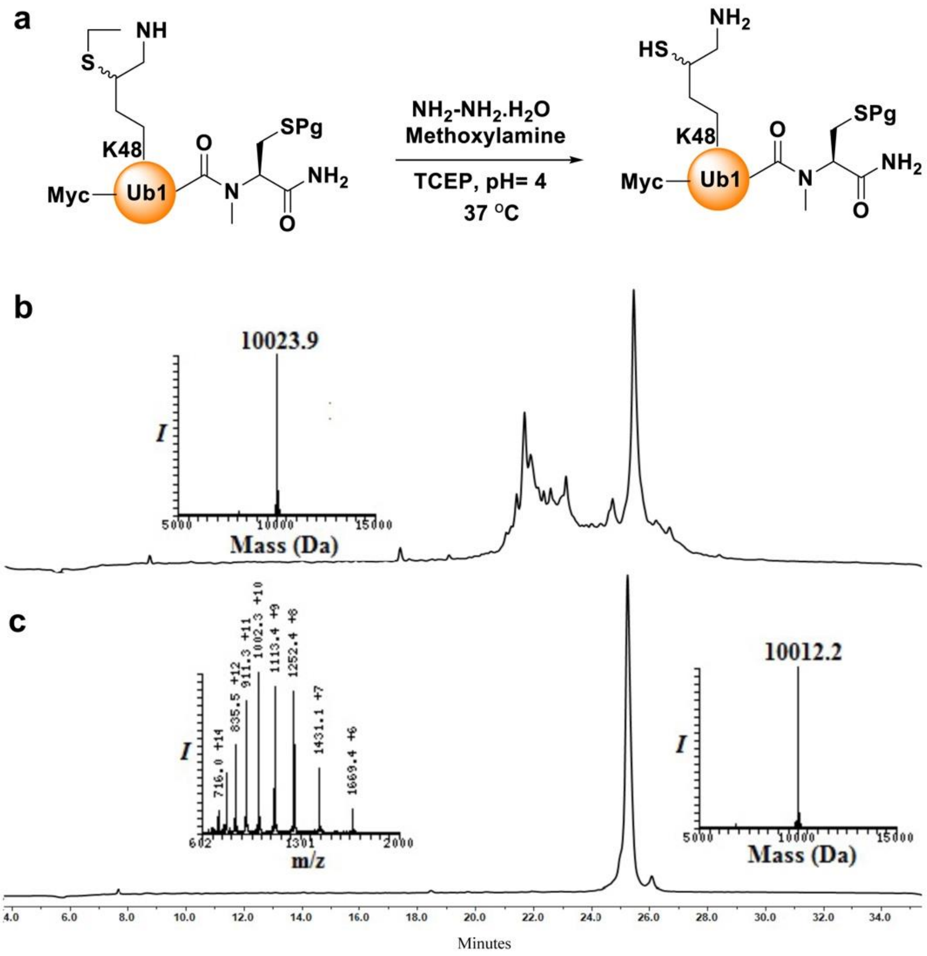

Supplementary Fig 25: Synthesis of Myc-Ub. **a**, Scheme depicts synthesis of Myc-Ub1 building block. **b**, Analytical HPLC and mass traces of the crude peptide – Myc-Ub1 (building block with δ−mercaptolysine at K48); peak *a* corresponds to the desired product with the observed mass of 10023.9 Da (calculated 10027.1 Da). **c**, Analytical HPLC and mass traces of purified thz deprotected Myc-Ub1 after methoxylamine treatment with the observed mass 10012.2 Da (calculated 10015.1 Da).

Synthesis of Ub2 and Ub3

a) Conversion of Ub2K48-N-methyl cysteine to 3-mercaptopropionic acid ester

Ub2K48-N-methyl cysteine (50 mg) was dissolved in 6 M Gn·HCl buffer (2.82 mL, 2 mM) and was subjected for the UV irradiation (365 nm, 2 h) to remove photolabile 2-nitrobenzyl protecting group of the C-terminal Cys, followed by the pH adjustment (~1.5-2) and incubation with 20% (vol/vol) 3-mercaptopropionic acid (MPA) at 42^{0}C for 20 h. The reaction was followed by HPLC using a C18 analytical column and a gradient of 0-60% B over 30 min and LC-MS analysis. For preparative HPLC, the same gradient was used to afford the Ub2K48-MPA thioester at a yield of ∼10% (∼4.9 mg).

b) Thz deprotection of Ub3K48-N-methyl cysteine

Similar procedure used for the thz deprotection of Myc-UbK48-N-Methyl cysteine was used for thz deprotection of Ub3K48-N-methyl cysteine to yield the product in 9% yield (~4.5 mg).

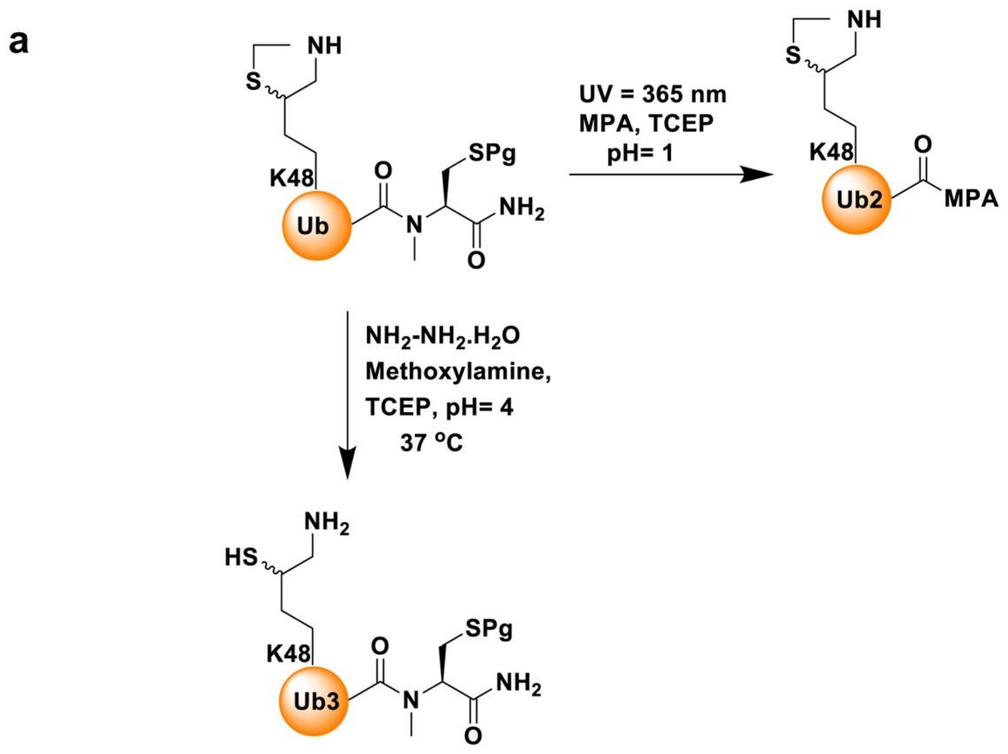

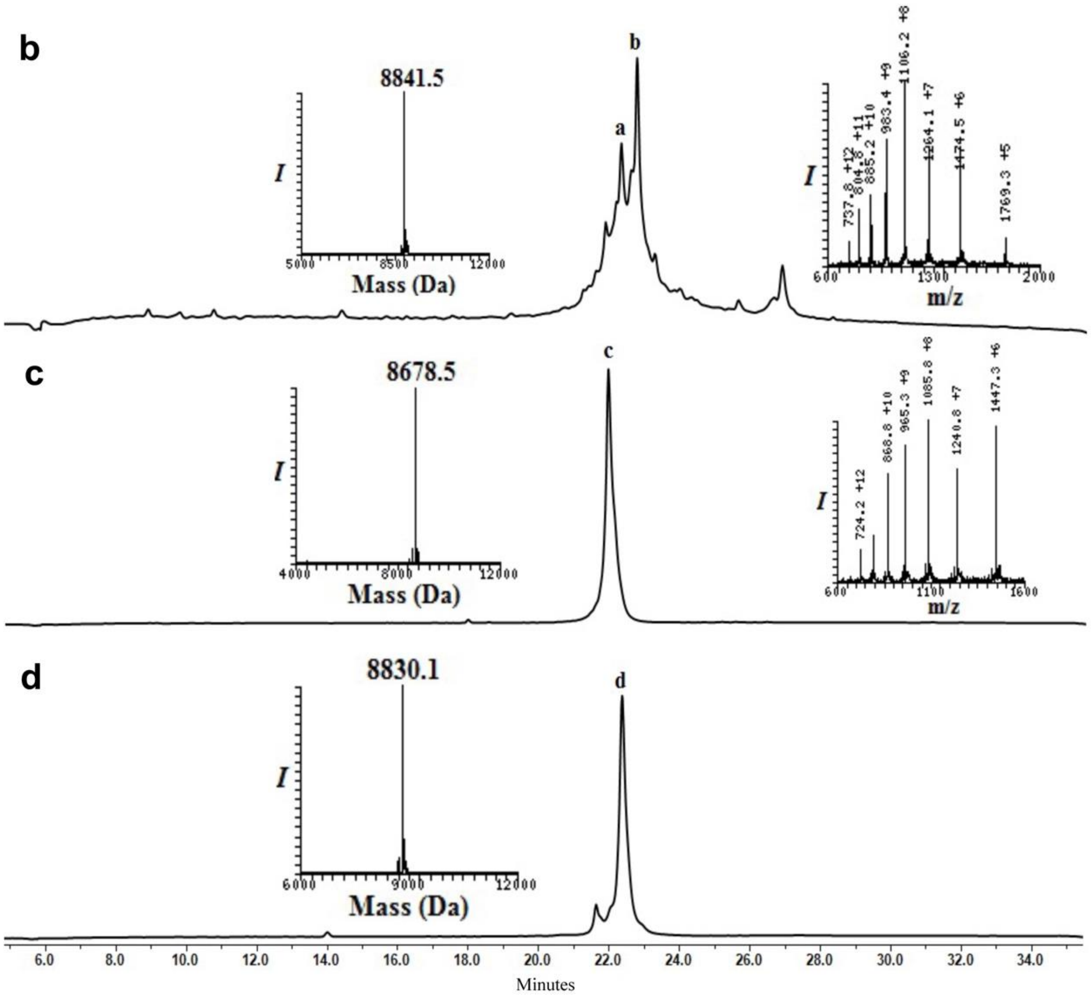

Supplementary Fig. 26: Synthesis of Ub2 and Ub3 building block with δ−mercaptolysine at K48. **a**, Scheme depicts synthesis of Ub2 and Ub3 building blocks, (Pg = O-Nitro Benzyl, MPA = 3-mercaptopropionic acid. **b**, Analytical HPLC and mass traces of the crude peptide of UbK48-N-Methyl cysteine; peak *b* corresponds to the desired product with the observed mass of 8841.5 Da (calculated 8,841.8 Da) peak *a* corresponds to deletion of arginine from Ub sequence. **c**, Analytical HPLC and mass traces of the purified Ub2-MPA thioester after N-methyl cysteine switching to MPA thioester with the observed mass 8678.5 Da (calculated 8,678.8 Da). **d**, Analytical HPLC and mass traces of the purified thz deprotected Ub3 after methoxylamine treatment with observed mass 8830.1 (calculated 8,830.8 Da).

Synthesis of Flag-Ub

DYKDDDDKNleQIFVKTLTGKTITLEVEPSDTIENVKAKIQDKEGIPPDQQRLIFAGKQ LEDGRTLSDYNIQKESTLHLVLRLRGG-*N*-Methyl Cysteine

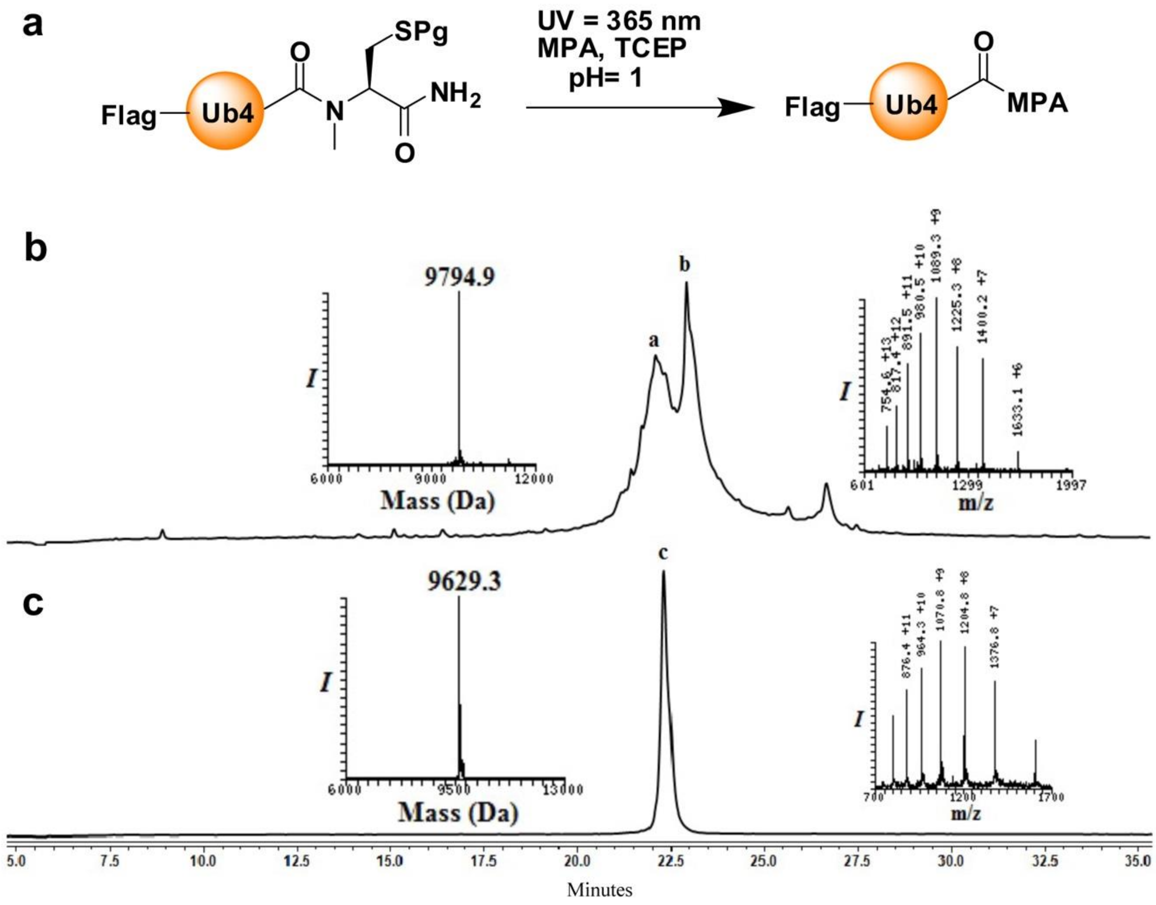

Supplementary Fig. 27: Synthesis of Flag-Ub. a, Synthesis of Flag-Ub4-MPA, (Pg = O-Nitro Benzyl, MPA = 3-mercaptopropionic acid). **b**, Analytical HPLC and mass traces of the crude Flag-Ub4-MPA building block (Flag-Ub4-N-Methyl cysteine); peak *b* corresponds to the desired product with the observed mass of 9794.9 Da (calculated 9792.7 Da) peak *a* with unresolved mass; **c**, Analytical HPLC and mass traces of the purified Flag-Ub4-MPA thioester after N-methyl cysteine switching to MPA thioester with the observed mass 9629.3 Da (calculated 9,630.7 Da).

Synthesis of MycUb-HA-CyclinB1-NT

Ligation of HA-CyclinB1-NT and MycUb1-MPA was carried out similar fashion as mentioned for conjugate **3** and product was subjected for the desulfurization to yield 40% of MycUb-HA-CyclinB1-NT.

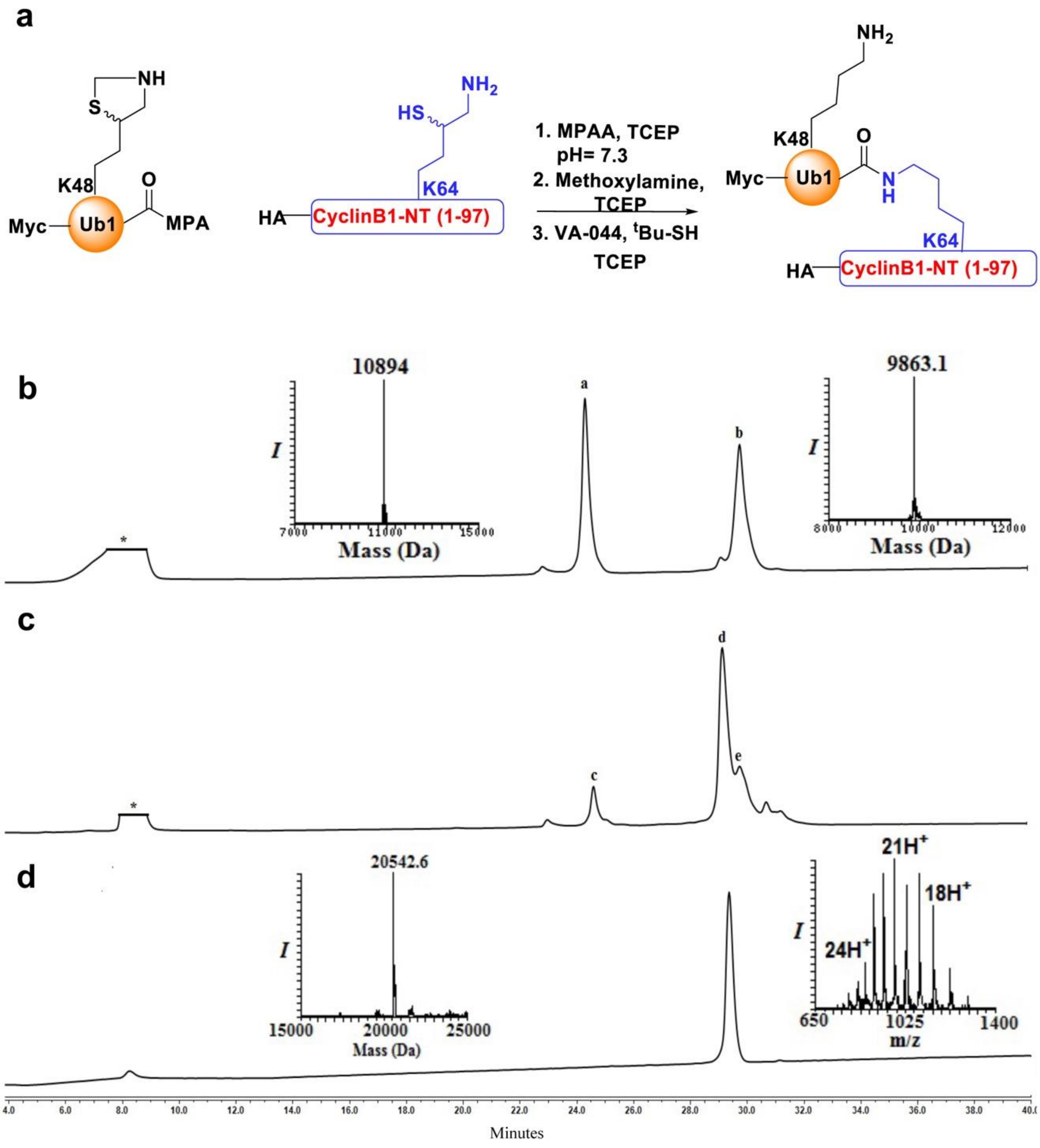

Supplementary Fig. 28: Synthesis of Myc-Ub-HA-CyclicB1-NT. **a**, Scheme depicting the ligation of HA-CyclinB1-NT (ThzOpen) and MycUbK48-MPA and synthesis of Myc-Ub-HA-CyclicB1-NT. **b**, Analytical HPLC and mass traces at ligation time zero, peak *a* corresponds to HA-CyclinB1-NT (ThzOpen), peak *b* corresponds to MycUbK48-MPA. **c**, Analytical HPLC and mass traces at ligation time 6 h, peak *c* corresponds to remaining of HA-CyclinB1-NT

(ThzOpen), peak *d* corresponds to the ligation product, peak *e* corresponds to the hydrolyzed MycUbK48-MPA. **d**, Desulfurized and Purified MycUb-HA-CyclinB1-NT with observed mass 20542.6 Da (Calculated 20543.6 Da).

Synthesis of Myc-DiUb^{K48}-MPA thioester, 4

Ligation: Thz deprotected MycUb1(K48*)-N-Me-Cysteine (10 mg, 1 mmol) and Ub2(K48)-MPA (10.4 mg, 1.2 mmol), were dissolved in 6 M Gn·HCl buffer (500 µL, 2 mM). To this solution, 20 equivalents each of MPAA and TCEP were added, the pH was adjusted to 7.3 and kept at room temperature for 30min and then at 37 ^{0}C for 4 h. The reaction was followed using analytical HPLC (C4 column) and a gradient of 5-55% B over 60 min. For preparative HPLC, the same gradient was used to isolate the ligation product in 52% yield (~9.7 mg). The conversion of MycDiUb–N-methyl cysteine to MycDiUb–MPA thioester was achieved by first removing the photolabile 2-nitrobenzyl protecting group of the C terminal Cys (365 nm, 2 h), followed by adjustment of pH ~1 and incubation with 20% (vol/vol) MPA at 42 ^{0}C for 20 h. The reaction was followed by HPLC using a C4 analytical column and a gradient of 5–55% B over 60 min and LC-MS analysis. For preparative HPLC, the same gradient was used to afford the Myc-DiUb^{K48}-MPA thioester, **4** at a yield of ∼38% (∼3.6 mg).

a

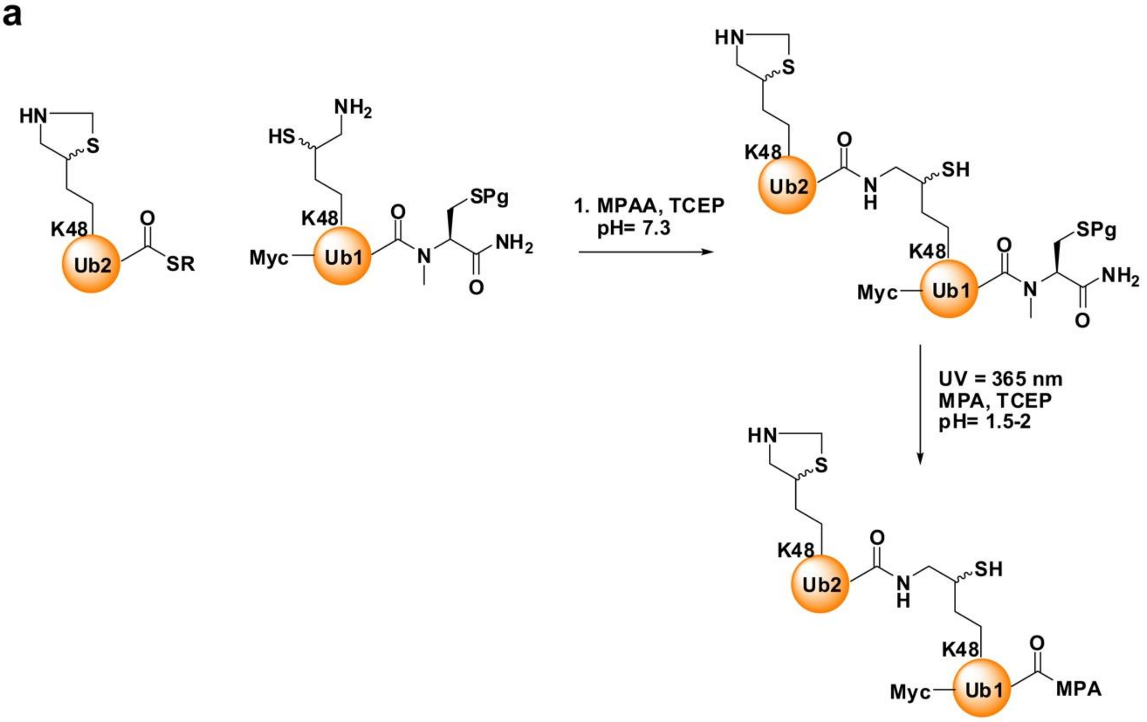

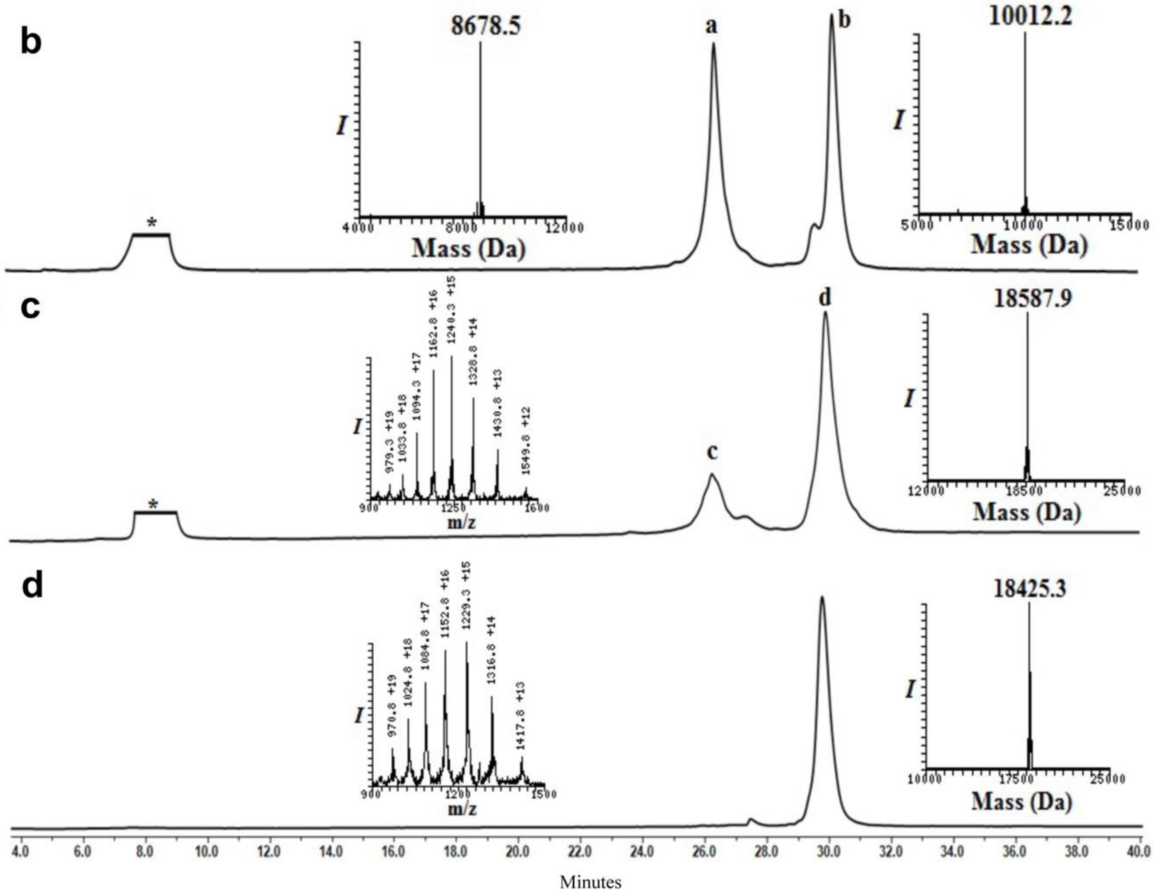

Supplementary Fig. 29: Synthesis of Myc-DiUb^{K48}-MPA. **a**, Scheme depicts synthesis of Myc-DiUb^{K48}-MPA, **4** (Pg = O-Nitro Benzyl, SR = 3-mercaptopropionic acid. **b**, Analytical HPLC and mass traces of the ligation at 0 h, peak *a* corresponds to Ub2K48-MPA, peak *b* corresponds to MycUb1(K48*)-N-methyl cysteine. **c**, Ligation after 6 h; peak *c* corresponds to the hydrolyzed Ub2K48-MPA thioester, peak *d* corresponds to the ligation product, Myc-DiUb^{K48}-N-Me-Cysteine with the observed mass of 18,587.9 Da (calculated 18,588.9 Da). **d**, Analytical HPLC and mass traces of purified Myc-DiUb^{K48}-MPA after N-Me-Cysteine switching to MPA reaction with observed mass 18425.3 Da (calculated 18425.9 Da)

Ligation of HA-CyclinB1-NT (ThzOpen), 3 with Myc-DiUb^{K48}-MPA, 4

HA-CyclinB1-NT (ThzOpen) (2 mg, 0.18 mmol) and Myc-DiUb^{K48}-MPA (3.6 mg, 0.19 mmol), were dissolved in 6 M Gn·HCl buffer (92 µL, 2 mM). To this solution, 20 equivalents each of MPAA and TCEP were added, the pH was adjusted to 7.3 and kept at 37 ^{0}C for 6 h. The reaction was followed using analytical HPLC (C4 column) and a gradient of 5-55% B over 60 min. After completion of ligation, Myc-DiUb^{K48}-HA-CyclinB1-NT was subjected for the Thz deprotection using hydrazine hydrate (80% solution, 100 equivalents) and kept at 25^{0}C for 1 h (pH ~7). To the above reaction mixture, methoxylamine (0.2 M, 15 equivalents) and tris-(2-carboxyethyl)-phosphine (TCEP, 30 equivalents) were added and incubated at 37 ^{0}C for 12 h to unmask the δ-mercaptolysine completely. The reaction was monitored using an analytical HPLC (C4 column) and a gradient of 5-55% B over 40 min and LC-MS analysis. For HPLC purification, a similar gradient was used to afford thz deprotected Myc-DiUb^{K48}-HA-CyclicB1-NT, **5** in ~40% yield (2.1 mg).

a

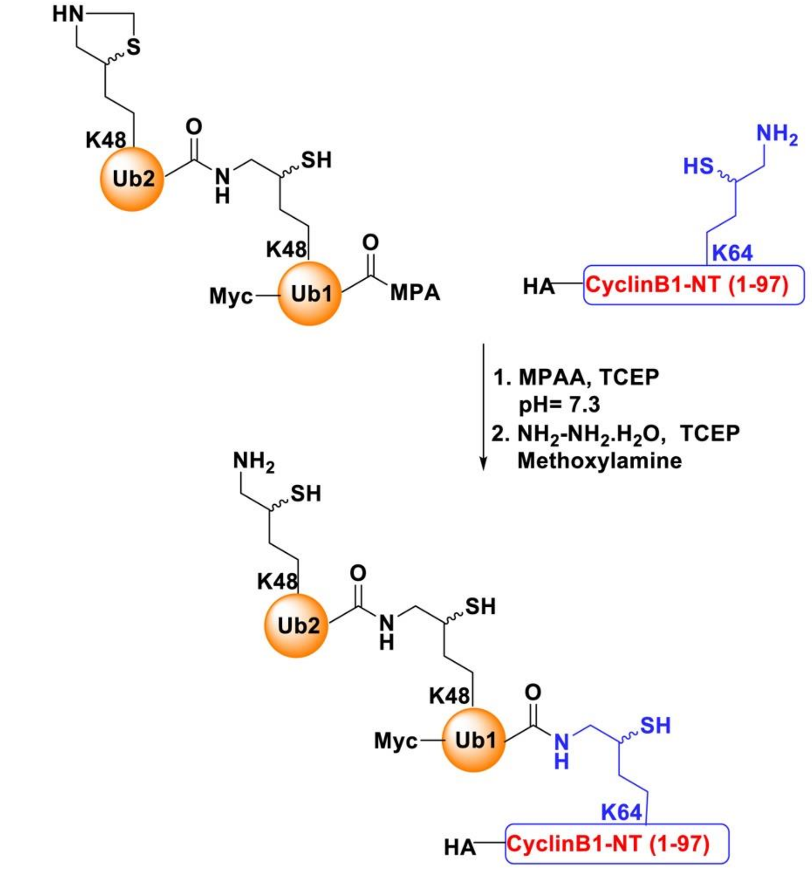

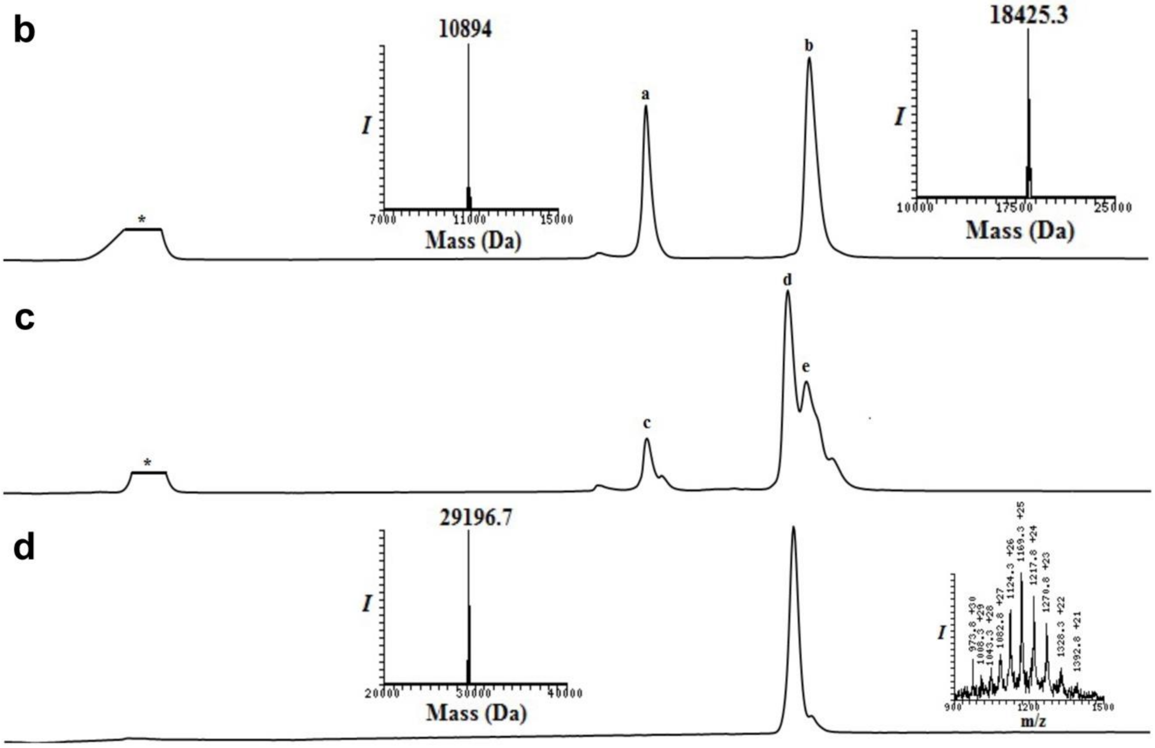

Supplementary Fig. 30: Synthesis of Myc-DiUb^{K48}-HA-CyclinB1-NT. **a**, Scheme depicts synthesis of Myc-DiUb^{K48}-HA-CyclinB1-NT. **b**, Analytical HPLC and mass traces of the ligation at 0 h, peak *a* corresponds to HA-CyclinB1-NT, peak *b* corresponds to Myc-DiUb^{K48}-MPA. **c**, Ligation after 6 h; peak *c* corresponds to the remaining HA-CyclinB1-NT, peak *d* corresponds to the ligation product, peak *e* corresponds to hydrolyzed Myc-DiUb^{K48}-MPA.**d**, Analytical HPLC and mass traces of Thz deprotected and purified Myc-DiUb^{K48}-HA-CyclinB1-NT with the observed mass of 29,196.7 Da (calculated 29197.3 Da).

Desulfurization of Myc-DiUb^{K48}-HA-CyclinB1-NT

Desulfurization of Myc-DiUb^{K48}-HA-CyclinB1-NT was carried out in similar fashion as mentioned for the conjugate **3,** to yield 35% of Myc-DiUb^{K48}-HA-CyclinB1-NT.

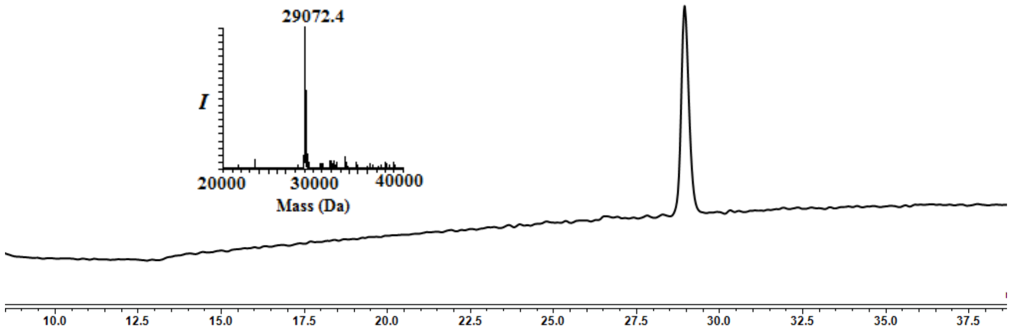

Supplementary Fig. 31: Analytical HPLC and mass traces for the desulfurized and purified Myc-DiUb^{48}-HA-CyclinB1-NT with the observed mass 29072.4 Da (calculated 29072.3 Da)

Synthesis of Flag-DiUb^{K48}-MPA, 6

Synthesis of Flag-DiUb^{K48}-MPA was carried out in similar way as described for the conjugate **4** to yield 35% (3.1mg)

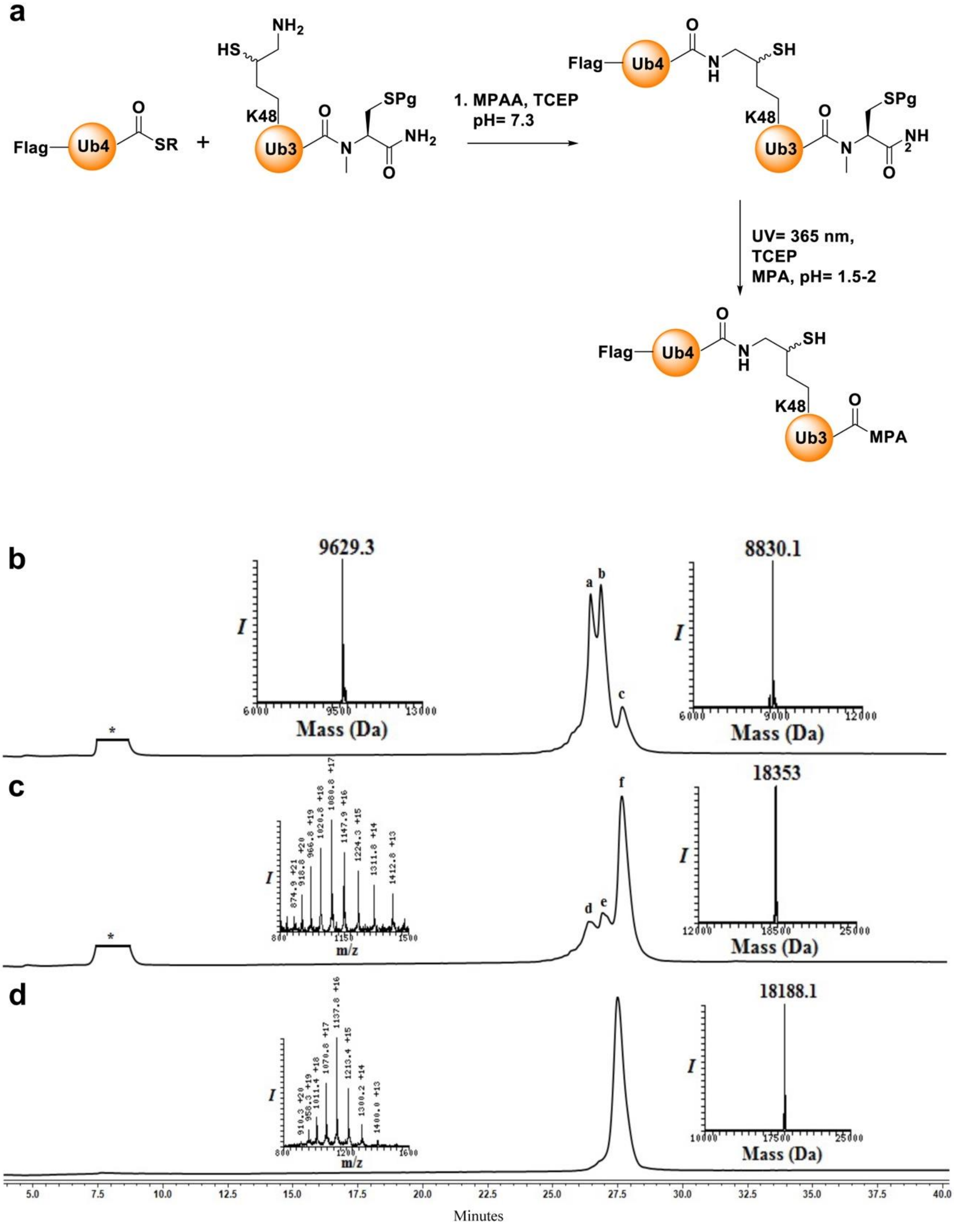

Supplementary Fig. 32: Synthesis of Flag-DiUb^{K48}-MPA. a, Scheme depicts synthesis of Flag-DiUb^{K48}-MPA, 6. **b**, Analytical HPLC and mass traces of the ligation at 0 h, peak *a* corresponds to Flag-Ub4-MPA, peak *b* corresponds to Ub3(K48*)-N-methyl cysteine, peak *c* corresponds to ligated product. **c**, Ligation after 6 h; peak *d* corresponds to the hydrolyzed Flag-Ub4-MPA thioester, peak *e* corresponds to remaining Ub3K48-N-methyl cysteine, peak *f* corresponds to the ligation product Flag-DiUb^{K48}-N-Methyl cysteine with the observed mass of 18,353 Da (calculated 18,354.5 Da). **d**, Analytical HPLC and mass traces of purified Flag-DiUb^{K48}-MPA with observed mass 18188.1 Da (calculated 18191.5 Da).

Ligation of Myc-DiUb^{K48}(Thz Open)-HA-CyclinB1-NT, 5 with Flag-DiUb^{K48}-MPA, 6

4 and **7** were ligated in a similar fashion as described for the synthesis of conjugate 8 and subjected for the desulfurization using VA-044 (20 equivalents, 0.1 M), TCEP (0.25 M) in the presence of 10% t-BuSH (v/v) at 37^{0}C for 18 h. The reaction was followed using analytical HPLC (C4 column) and a gradient of 5-55% B over 1hr. For semi-preparative HPLC, the same gradient was used to afford the desulfurized TetraUb^{K48}-HA-CyclinB1-NT ~30% yield (~1.6 mg).

a

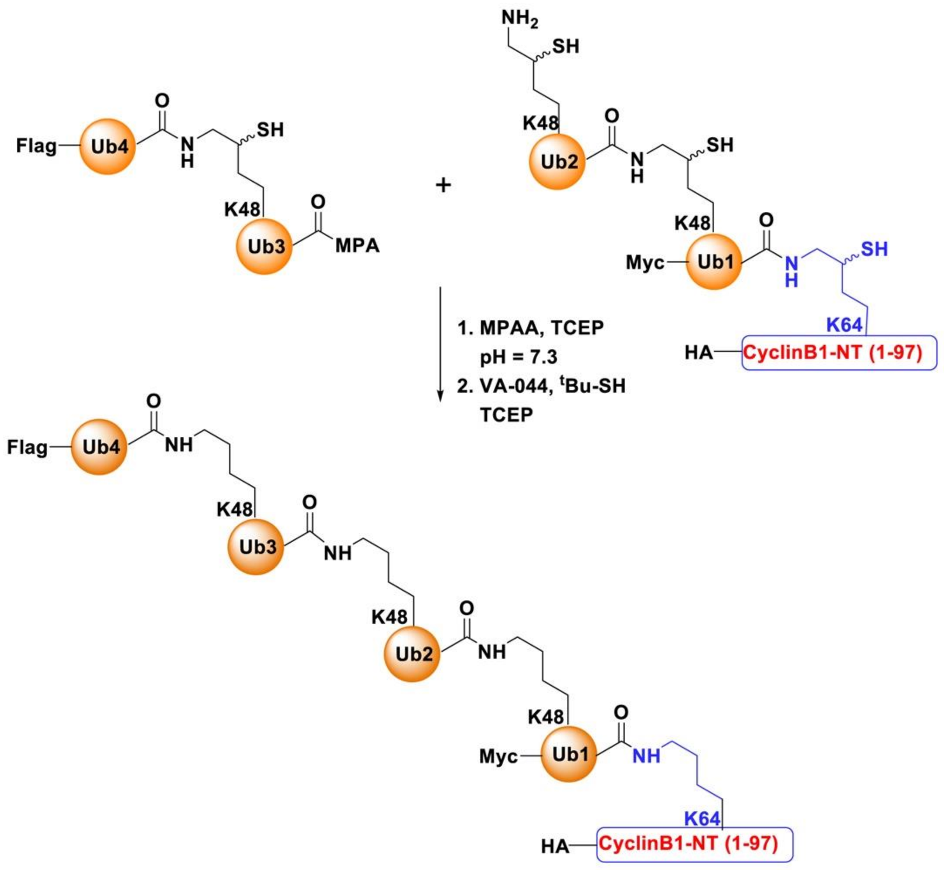

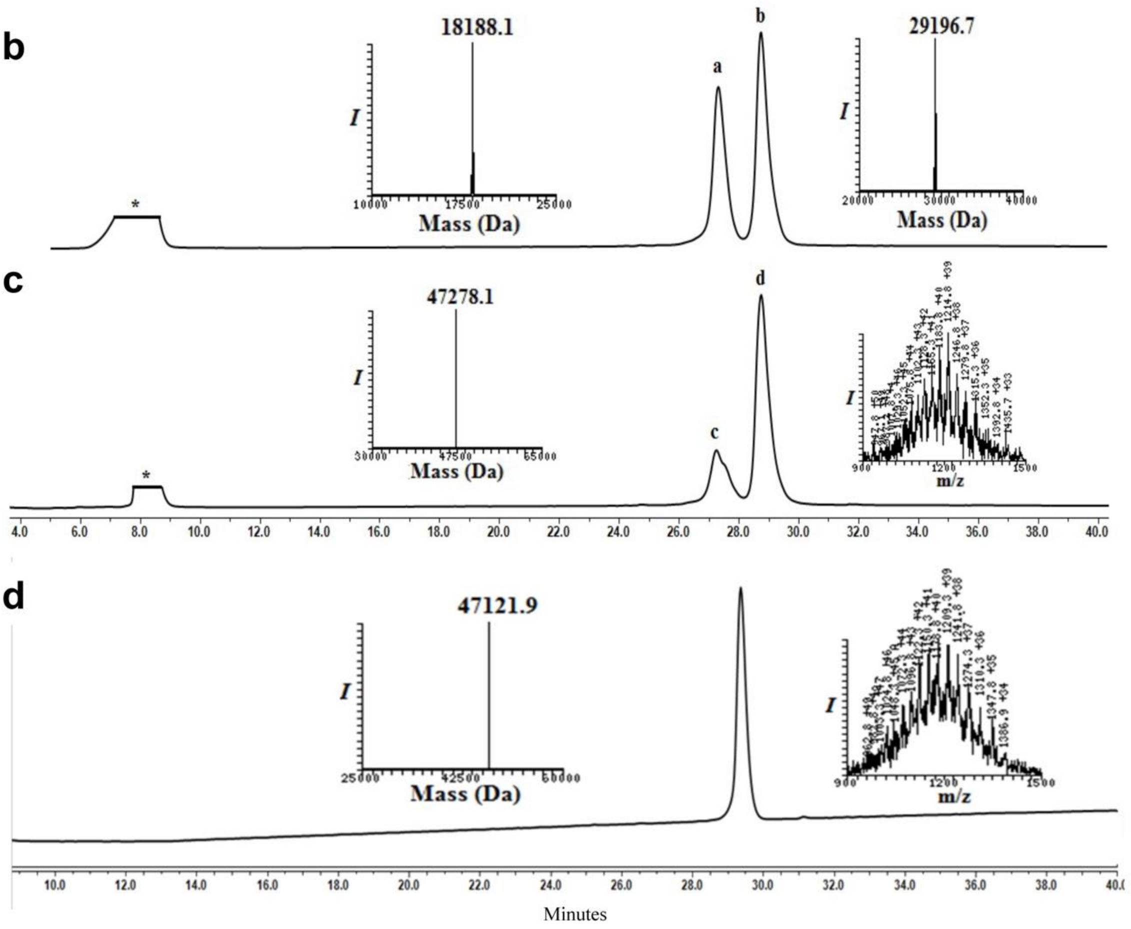

Supplementary Fig. 33: Synthesis of TetraUb^{K48}-HA-CyclinB1-NT. **a**, Scheme depicts synthesis of TetraUb-HA-CyclinB1-(NT). **b**, Analytical HPLC and mass traces of the ligation at 0 h, peak *a* corresponds to Flag-DiUb^{K48}-MPA, peak *b* corresponds to Myc-DiUb^{K48}-HA-CyclinB1-NT. **c**, Ligation after 6hrs; peak *c* corresponds to the hydrolyzed Flag-DiUb^{K48}-MPA thioester, peak *d* corresponds to ligation product TetraUb^{K48}-HA-CyclinB1-NT with the observed mass of 47278.1 Da (calculated 47279.9 Da). **d,** Analytical HPLC and mass traces for the desulfurized and purified TetraUb^{K48}-HA-CyclinB1-NT with the observed mass 47121.9 Da (calculated 47124.9 Da).

Strategy for expression of HA-CyclinB1-NT in mammalian cells

Expression of HA-CyclinB1-NT in HEK293 cells

When HA-cyclinB1-NT was expressed through pCMV10 plasmid in HEK293 cells, we could not detect any protein expression although there was expression of the mRNA. Since, CyclinB1-NT (1-88 aa) is an unstructured polypeptide, it most likely degrades rapidly after translation. Hence, in order to provide a stability as well as a marker for its protein expression we attached a self-immolating tag "NS3-protease" with its own cleavage site in between NS3-domain and HA-CyclinB1-NT as described in **Fig. 5a**. Post-translation of this chimeric protein, NS3 tag is removed by auto cleavage and allow HA-CyclinB1-NT to be degraded by proteasome.

Expression of K0 mutant of HA-CyclinB1-NT in HEK293 cells

When the K0 mutant of HA-cyclinB1-NT was expressed with the NS3-tag we could not detect its protein levels due to rapid degradation by proteasome (**Supplementary Fig. 11b**). In order to track its rate of degradation we follow a "bortezomib pulse-chase" experiment where we first accumulated K0-HA-CyclinB1-NT proteins by 6 h treatment of bortezomib (500nM) then chased the rate of CyclinB1 degradation after removal of bortezomib from media. We did also confirm that inhibition of proteasome by bortezomib in cells is reversible and by 6h 50% of proteasome activity was restored. (**Supplementary Fig. 11c & d**).

DNA sequence for cloning of HA-CyclinB1-NT

DNA Fragment 1: EcoRI-Kozak Sequence-ATG-3XFLAG-Linker-NS3Pro-NS5A5B cleavage site-BamHI.

DNA sequence:

5’gaattcgccaccatggactacaaagaccatgacggtgattataaagatcatgacatcgattacaaggatgacgatgacaagatggc
gcccatcacggcgtacgcccagcagacgagaggcctcctagggtgtataatcaccagcctgactggccgggacaaaaaccaagtg
gagggtgaggtccagatcgtgtcaactgctacccaaaccttcctggcaacgtgcatcaatggggtatgctgggcagtctaccacggg
gccggaacgaggaccatcgcatcacccaagggtcctgtcatccagatgtataccaatgtggaccaagaccttgtg ggctggcccgct
cctcaaggttcccgctcattgacaccctgtacctgcggctcctcggacctttacctggtcacgaggcacgccgatgtcattcccgtgcg
ccggcgaggtgatagcaggggtagcctgctttcgccccggcccatttcctacttgaaaggctcctcggggggtccgctgttgtgcccc
gcgggacacgccgtgggcctattcagggccgcggtgtgcacccgtggagtggctaaagcggtggactttatccctgtggagaaccta
gagacaaccatgagatccccggtgttcacggacgtgggcaggatcgtcttgtccgggaagccggcaggcagtagcggaagcagtat
tatacctgacagggaggttctctaccaggagttcgaagatgtcgtgccatgctcaatgggatcc 3’

DNA Fragment 2: BamHI- HA-Cyclin B1-NT-HindIII

DNA Sequence (WT-CyclinB1-NT): 5’
ggatccatgtatccatatgatgttccagattatgctatggcgctccgagtcaccaggaactcgaaaattaatgctgaaaataaggcgaag
atcaacatggcaggcgcaaagcgcgttcctacggcccctgctgcaacctccaagcccggactgaggccaagaacagctcttgggga
cattggtaacaaagtcagtgaacaactgcaggccaaaatgcctatgaagaaggaagcaaaaccttcagctactggaaaa gtcattgat
aaaaaactaccaaaacctcttgaaaaggtacctatgtgaaagctt 3’

DNA Sequence (K0-CyclinB1-NT): 5’
ggatccatgtatccatatgatgttccagattatgctctcgcgctccgagtcaccaggaactcgcgcattaatgctgaaaatcgggcgcgc
atcaacatggcaggcgcacgtcgcgttcctacggcccctgctgcaacctcccgccccggactgaggccaagaac agctcttgggga
cattggtaaccgcgtcagtgaacaactgcaggcccggatgcctatgcgcagagaagcacgaccttcagctactggacgcgtcattgat
cggcgcctaccacgccctcttgaaagagtacctctctgaaagctt 3’
