# Supplementary material for: The 20S as a stand-alone proteasome in cells can degrade the ubiquitin tag
Source: Nat Commun. 2021 Oct 26;12:6173. doi: 10.1038/s41467-021-26427-0 (PMC8548400; doi:10.1038/s41467-021-26427-0)
Supplement: Supplementary file 3 — Supplementary Data legends [file 41467_2021_26427_MOESM3_ESM.docx]

**Supplementary Data legends**

**The 20S as a stand-alone proteasome in cells can degrade the ubiquitin-tag**

Indrajit Sahu^1^, Sachitanand M. Mali^2^, Prasad Sulkshane^1^, Cong Xu^3^, Andrey Rozenberg^1^, Roni Morag^1^, Manisha Priyadarsini Sahoo^1^, Sumeet K. Singh^2^, Zhanyu Ding^3^, Yifan Wang^3^, Sharleen Day^4^, Yao Cong^3,5^, Oded Kleifeld^1,*^, Ashraf Brik^2,*^, Michael H. Glickman^1,*^

*Correspondence to: [glickman@technion.ac.il](mailto:glickman@technion.ac.il), [okleifeld@technion.ac.il](mailto:okleifeld@technion.ac.il), or [abrik@technion.ac.il](mailto:abrik@technion.ac.il)

**File Name:** Supplementary Data 1

**Description:** **MS/MS analysis of purified proteasomes.** The purified 26S and 20S proteasomes were run in native gels followed by detection with in-gel activity assay (See method section). The corresponding active bands were excised from the gel and subjected to peptide extraction and MS/MS analysis. The LFQ intensity and MS/MS count was calculated for each peptide identified for each proteasome subunits. (Related to Supplementary Fig 1c).

**File Name:** Supplementary Data 2

**Description:** **MS/MS analysis of degraded products of CyclinB1-NT from the 20S and 26S proteasomes.** The degraded products of unmodified CyclinB1-NT from the 20S and 26S proteasome reactions were subjected to non-tryptic MS/MS analysis and the raw data were analyzed by TPP software (See Method section). (Related to Fig. 2g and 2h).

**File Name:** Supplementary Data 3

**Description:** **MS/MS analysis of degraded products of MonoUb-CyclinB1-NT from the 20S and 26S proteasomes.** The degraded products of MonoUb-CyclinB1-NT from the 20S and 26S proteasome reactions were subjected to non-tryptic MS/MS analysis and the raw data were analyzed by TPP software (See Method section). (Related to Fig. 2g and 2h).

**File Name:** Supplementary Data 4

**Description:** **MS/MS analysis of degraded products of TetraUb-CyclinB1-NT from the 20S and 26S proteasomes.** The degraded products of TetraUb-CyclinB1-NT from the 20S and 26S proteasome reactions were subjected to non-tryptic MS/MS analysis and the raw data were analyzed by TPP software (See Method section). (Related to Fig. 2g and 2h).

**File Name:** Supplementary Data 5

**Description:** **MS/MS analysis of degraded ubiquitin tags of MonoUb-CyclinB1-NT and TetraUb-CyclinB1.** The degraded products of MonoUb-CyclinB1-NT and TetraUb-CyclinB1 from the 20S proteasome reactions were subjected to non-tryptic MS/MS analysis and the raw data were analyzed by MaxQuant (See Method section). The LFQ intensity and MS/MS count was calculated for each peptide derived from the ubiquitin tags (Related to Fig 3c).

**File Name:** Supplementary Data 6

**Description:** **MS/MS analysis of ubiquitin remnants from MonoUb-CyclinB1-NT and DiUb-CyclinB1.** The degraded products of MonoUb-CyclinB1-NT and DiUb-CyclinB1 from the 20S proteasome reactions were subjected to non-tryptic MS/MS analysis and the raw data were analyzed by MSFragger (See Method section). (Related to Fig 3d and Supplementary Table 1).

**File Name:** Supplementary Data 7

**Description:** **MS/MS analysis of the intracellular peptides from PSMD2-KD (Hi20S) cells.** The intracellular peptides from the control cells and PSMD2-KD (Hi20S) cells were isolated (see Method section) and subjected to non-tryptic MS/MS analysis. The raw data was analyzed by MaxQuant. (Related to Fig 6 and Supplementary Fig 13).

**File Name:** Supplementary Data 8

**Description:** **MS/MS analysis of the intracellular peptides from PSMD2-KD (Hi20S) cells.** The intracellular peptides from the control cells and PSMD2-KD (Hi20S) cells were isolated (see Method section) and subjected to non-tryptic MS/MS analysis. The raw data was analyzed by TPP. (Related to Fig 6 and Supplementary Fig 13).

**File Name:** Supplementary Data 9

**Description:** **MS/MS analysis of the intracellular peptides from HeLa Hypoxic cells.** The intracellular peptides from HeLa cells at both normoxia and hypoxia conditions were isolated (see Method section) and subjected to non-tryptic MS/MS analysis. The raw data was analyzed by MaxQuant. (Related to Fig 8b and 8c).

**File Name:** Supplementary Data 10

**Description:** **MS/MS analysis of the intracellular peptides from Failing heart.** The intracellular peptides from the Normal and Failing heart samples were isolated (see Method section) and subjected to non-tryptic MS/MS analysis. The raw data was analyzed by MaxQuant. (Related to Fig 8f and Supplementary Fig 16c, d).

**File Name:** Supplementary Data 11

**Description:** **MS/MS analysis of the intracellular peptides from Failing heart.** The intracellular peptides from the Normal and Failing heart samples were isolated (see Method section) and subjected to non-tryptic MS/MS analysis. The raw data was analyzed by TPP. (Related to Fig 8g and Supplementary Fig 18a, b).
