# Supplementary material for: The 20S as a stand-alone proteasome in cells can degrade the ubiquitin tag
Source: Nat Commun. 2021 Oct 26;12:6173. doi: 10.1038/s41467-021-26427-0 (PMC8548400; doi:10.1038/s41467-021-26427-0)
Supplement: Supplementary file 15 — Reporting Summary [file 41467_2021_26427_MOESM15_ESM.pdf]

nature research

Corresponding author(s): Michael H. Glickman, Ashraf Brik, and Oded Kleifeld

Last updated by author(s): Oct 1st, 2021

Reporting Summary

Nature Research wishes to improve the reproducibility of the work that we publish. This form provides structure for consistency and transparency in reporting. For further information on Nature Research policies, see our Editorial Policies and the Editorial Policy Checklist.

Statistics

For all statistical analyses, confirm that the following items are present in the figure legend, table legend, main text, or Methods section.

n/a	Confirmed	
☐	☒	The exact sample size (*n*) for each experimental group/condition, given as a discrete number and unit of measurement
☐	☒	A statement on whether measurements were taken from distinct samples or whether the same sample was measured repeatedly
☐	☒	The statistical test(s) used AND whether they are one- or two-sided<br>*Only common tests should be described solely by name; describe more complex techniques in the Methods section.*
☐	☒	A description of all covariates tested
☐	☒	A description of any assumptions or corrections, such as tests of normality and adjustment for multiple comparisons
☐	☒	A full description of the statistical parameters including central tendency (e.g. means) or other basic estimates (e.g. regression coefficient) AND variation (e.g. standard deviation) or associated estimates of uncertainty (e.g. confidence intervals)
☐	☒	For null hypothesis testing, the test statistic (e.g. *F*, *t*, *r*) with confidence intervals, effect sizes, degrees of freedom and *P* value noted<br>*Give P values as exact values whenever suitable.*
☒	☐	For Bayesian analysis, information on the choice of priors and Markov chain Monte Carlo settings
☒	☐	For hierarchical and complex designs, identification of the appropriate level for tests and full reporting of outcomes
☒	☐	Estimates of effect sizes (e.g. Cohen's *d*, Pearson's *r*), indicating how they were calculated

Our web collection on statistics for biologists contains articles on many of the points above.

Software and code

Policy information about availability of computer code

Data collection	The CryoEM images of proteasomes were collected by Titan Krios transmission electron microscope (Thermo Fisher) utilizing the SerialEM v3.7 automated data collection software package.<br>Mass-spectrometry data was collected using either Q-Exacitive-Plus/Q-Exactive HF mass spectrometer (Thermo Fisher) or Orbitrap Fusion Tribrid (Thermo Scientific) coupled to Ultimate 3000 Nano Systems (Thermo Scientific).<br>The real-time PCR data was collected by Applied Biosystems QuantStudio 1 system.
Data analysis	Single particle CryoEM data analysis was executed in RELION 3.0 software, and images were aligned and summed using MotionCor2 software. After CTF parameter determination downstream data processing was carried out using CTFFIND4 and Gctf. Model building was performed by flexible fitting of to the proteasome CryoEM model against the corresponding electron density map using the real-space refinement function in Rosetta. The generated model was further refined utilizing PHENIX, and manually modified using COOT. UCSF Chimera and ChimeraX were used for final image production. 3D variability analyses (3DVA) map was generated using CryoSparc v3.2.<br>Mass-spectrometry data analysis was performed using either MaxQuant version 1.6.7.0 or the Trans Proteomic Pipeline (TPP) v5.2.0 Flammagenitus softwares. The raw files were searched against the Homo sapiens UniProt FASTA database (November 2017; 20,239 sequences). TPP searches were done following RAW files conversion to mzML using MSConvert (ver 3.01157) using centroid and searches were performed using Comet (2017.01 rev. 1). Further data analysis was performed as follows: plotMDS function of edgeR v. 3.26, sequence logos using dagLogo v. 1.22.2 and DiffLogo v. 2.8.0, peptide size distribution by lme4 v. 1.1 and emmeans v.1.3.5, statistical tests were performed in R v.3.6.1, prediction of protein disorder was done using IUPRED2A, BoxPlotR 89 (http://shiny.chemgrid.org/boxplotr/), Graphpad Prism V5.<br>IB quantification was done using ImageJ software (Mac Biophotonics) and Graphpad Prism V5.<br>The realtime PCR data was analyzed using QuantStudio™ Design and Analysis Software (Thermo Fisher).

For manuscripts utilizing custom algorithms or software that are central to the research but not yet described in published literature, software must be made available to editors and reviewers. We strongly encourage code deposition in a community repository (e.g. GitHub). See the Nature Research guidelines for submitting code & software for further information.

Data

Policy information about availability of data

All manuscripts must include a data availability statement. This statement should provide the following information, where applicable:

- Accession codes, unique identifiers, or web links for publicly available datasets
- A list of figures that have associated raw data
- A description of any restrictions on data availability

All analyzed data are uploaded along with the manuscript as Supplementary data sheets and Source data compiling uncropped images and datapoints used in graphs . The MS proteomics data have been deposited to the ProteomeXchange Consortium via the PRIDE partner repository with the dataset identifier PXD018711 [https://www.ebi.ac.uk/pride/archive/projects/PXD018711] and PXD018722 [https://www.ebi.ac.uk/pride/archive/projects/PXD018722]. Cryo-EM maps determined from the 20S-alone dataset has been deposited at the Electron Microscopy Data Bank with accession codes EMD-13389 [https://www.ebi.ac.uk/pdbe/entry/emdb/EMD-13389] (before post-processing) and EMD-31730 [https://www.ebi.ac.uk/pdbe/entry/emdb/EMD-31730], (after post-processing by RELION), and associated atomic models have been deposited in the Protein Data Bank with accession code 7PG9. Cryo-EM maps determined from the 20S+monoUb-CyclinB1-NT dataset have been deposited at the Electron Microscopy Data Bank with accession codes of EMD-31728 [https://www.ebi.ac.uk/pdbe/entry/emdb/EMD-31728] (S0), EMD-31724 [https://www.ebi.ac.uk/pdbe/entry/emdb/EMD-31724] (S1), and EMD-31727 [https://www.ebi.ac.uk/pdbe/entry/emdb/EMD-31727] (S2), and related models have been deposited in the Protein Data Bank under accession code of 7V5G [http://doi.org/10.2210/pdb7V5G/pdb] (S1), 7V5M [http://doi.org/10.2210/pdb7V5M/pdb] (S2). There is no PDB entry corresponding to EMD-31728 (S0). Source data are provided with this paper.

Field-specific reporting

Please select the one below that is the best fit for your research. If you are not sure, read the appropriate sections before making your selection.

- [x] Life sciences
- [ ] Behavioural & social sciences
- [ ] Ecological, evolutionary & environmental sciences

For a reference copy of the document with all sections, see nature.com/documents/nr-reporting-summary-flat.pdf

Life sciences study design

All studies must disclose on these points even when the disclosure is negative.

Sample size	No sample size calculation was performed prior to experiments. However, in each case sample size was appropriate based on the consistency of measurable differences between groups. Essentially for statistical analysis we chose n of at least 3.
Data exclusions	No data were excluded from analysis.
Replication	All experiments performed in this study were reliably reproducible. Information about the number of replicates is indicated in the relevant figure legends.
Randomization	We have not performed any randomization techniques for any of our experiments since it was not applicable due to the nature of our study. Samples were distributed based on the genotypes/treatments/experimental conditions applied and their identity was known befor experimental set up and data analysis.
Blinding	The investigators were blinded for all Mass-spectrometry data collection and in a few cases for data analysis wherever necessary. For other cell biology, biochemical or structural experiments the groups/sets/samples can not be blinded since they are easily identifiable due to their distinct properties.

Reporting for specific materials, systems and methods

We require information from authors about some types of materials, experimental systems and methods used in many studies. Here, indicate whether each material, system or method listed is relevant to your study. If you are not sure if a list item applies to your research, read the appropriate section before selecting a response.

Materials & experimental systems

n/a	Involved in the study	
	x	Antibodies
	x	Eukaryotic cell lines
x		Palaeontology and archaeology
x		Animals and other organisms
	x	Human research participants
x		Clinical data
x		Dual use research of concern

Methods

n/a	Involved in the study	
x		ChIP-seq
x		Flow cytometry
x		MRI-based neuroimaging

Antibodies

Antibodies used	Mouse monoclonal anti-HA antibody, Sigma-SAB1305536-40TST<br>Rabbit monoclonal anti-HA-HRP R&D Systems-HAM0601<br>Mouse monoclonal anti-aplha6 *<br>Mouse monoclonal anti-FLAG, Biolegend-637301, Clone-L5<br>Mouse monoclonal anti-Myc, Biolegend-626802, Clone-9E10<br>Mouse monoclonal anti-Rpt5*<br>Rabbit polyclonal anti-PSMC4, proteintech-11389-1-AP<br>Mouse monoclonal anti-Rpn10*<br>Mouse monoclonal anti-PSMD1*<br>Rabbit polyclonal anti-PSMD2 Bethyl-A303-853A<br>Mouse monoclonal anti-beta3*<br>Mouse monoclonal anti-USP14, Sigma-WH0009097M4<br>Mouse monoclonal anti-ubiquitin, Covance-MMS-257P-200, Clone P4D1<br>Rabbit polyclonal anti-ubiquitin, Dako-Z0458<br>Rabbit monoclonal anti-ubiquitin K48-specific, Millipore-05-1307, Clone Apu2<br>Rabbit monoclonal anti-ubiquitin K63-specific, Millipore-05-1308, Clone Apu3<br>Rabbit polyclonal anti-CyclinB1 N-terminal, abcam-ab226397<br>Rabbit polyclonal anti-GAPDH, Sigma-G9545<br>Rabbit polyclonal anti-Mcl1, abcam-ab32087<br>Rabbit polyclonal anti-LC3B, abcam-ab51520<br>Mouse monoclonal anti-alpha tubulin, Biolegend-627902, Clone 10D8<br>Rabbit monoclonal [EPR16897] anti-HIF1alpha, abcam-ab179483<br>Mouse monoclonal anti-Puromycin, Millipore-MABE343, Clone 12D10<br>Rabbit polyclonal anti-PSME4(PA200) []EPR13577(B)]-C-terminal, abcam181203<br>Rabbit polyclonal anti-PA28alpha, Cell signaling-2408<br>Mouse secondary antibody-HRP, Jackson Immuno research-115-035-003<br>Rabbit secondary antibody-HRP, Santa Cruz-sc2004
Validation	The validation reports of all commercially available antibodies are provided in the database of manufacturer's website. The antibodies (with*) procured from other labs or generated in house are validated by immunoblotting using HEK293T cell lysates. Those antibodies are as follows:<br>Mouse monoclonal anti-alpha6: WB working dilutions 1:5000<br>Mouse monoclonal anti-PSMD2: WB working dilutions 1:5000<br>Mouse monoclonal anti-Rpn10: WB working dilutions 1:1000<br>Mouse monoclonal anti-beta3: WB working dilutions 1:5000<br>Mouse monoclonal anti-Rpt5: WB working dilutions 1:3000<br>Both Mouse secondary antibody-HRP and Rabbit secondary antibody-HRP are validated at dilutions 1:5000

Eukaryotic cell lines

Policy information about cell lines

Cell line source(s)	HEK293T and HeLa cell lines were purchased from ATCC. The T-47D-PSMD2 knock-down stable cell line was procured from Peter Tsvetkov, Broad Institute, US.
Authentication	Cell lines were not authenticated by ourselves.
Mycoplasma contamination	All cell lines were tested to be Mycoplasma negative.
Commonly misidentified lines (See ICLAC register)	Non of the cell lines used in the study were found in Commonly misidentified cell line database.

Human research participants

Policy information about studies involving human research participants

Population characteristics	Adult population, both male and female, unknown genotype, with advanced, end stage heart failure undergoing cardiac transplantation or donor hearts from brain dead organ donors. Informed consent from the family was provided.
Recruitment	There was no specific recruitment mechanism. Brain dead donor hearts were acquired through the Gift of Life Organ Procurement organization when the heart was not suitable for transplantation. The hearts from patients with heart failure were acquired as discarded tissue at the time of cardiac transplantation. Informed consent was provided.
Ethics oversight	University of Michigan, Ann Arbor, Michigan, USA

Note that full information on the approval of the study protocol must also be provided in the manuscript.
